# Pune GSH supplementation study: Analyzing longitudinal changes in type 2 diabetic patients using linear mixed-effects models

**DOI:** 10.3389/fphar.2023.1139673

**Published:** 2023-03-13

**Authors:** Arjun Kolappurath Madathil, Saroj Ghaskadbi, Saurabh Kalamkar, Pranay Goel

**Affiliations:** ^1^ Biology Division, Indian Institute of Science Education and Research, Pune, India; ^2^ Department of Zoology, Savitribai Phule Pune University, Pune, India

**Keywords:** GSH supplementation, type 2 diabetes, HbA1c, 8-OHdG, elderly diabetic population, mixed-effects models

## Abstract

Oral GSH supplementation along with antidiabetic treatment was shown to restore the body stores of GSH significantly and reduce oxidative DNA damage (8-OHdG) in Indian Type 2 diabetic (T2D) patients over 6 months in our recent clinical study. Post hoc analysis of the data also suggested that elder patients benefit from improved HbA1c and fasting insulin. We modeled longitudinal changes in diabetic individuals using a linear mixed-effects (LME) framework and obtained i) the distribution of individual trajectories with and without GSH supplementation and ii) the overall rates of changes in the different study arms. Serial changes in elder and younger diabetic individuals were also modeled independently to examine differences in their progression. The average linear trajectories obtained from the model explain how biochemical parameters in T2D patients progress over 6 months on GSH supplementation. Model estimates show improvements in erythrocytic GSH of 108 µM per month and a reduction in 8-OHdG at a rate of 18.5 ng/μg DNA per month in T2D patients. GSH replenishes faster in younger people than in the elder. 8-OHdG reduced more rapidly in the elder (24 ng/μg DNA per month) than in younger (12 ng/μg DNA per month) individuals. Interestingly, elder individuals show a substantial reduction in HbA1c (0.1% per month) and increased fasting insulin (0.6 µU/mL per month). Changes in GSH correlate strongly with changes in HbA1c, 8-OHdG, and fasting insulin in the elder cohort. The model estimates strongly suggest it improves the rate of replenishment in erythrocytic GSH stores and reduces oxidative DNA damage. Elder and younger T2D patients respond differently to GSH supplementation: It improves the rate of reduction in HbA1c and increases fasting insulin in elder patients. These model forecasts have clinical implications that aid in personalizing treatment targets for using oral GSH as adjuvant therapy in diabetes.

## Introduction

A large number of clinical and experimental studies have demonstrated the role of oxidative stress in developing type 2 diabetes (T2D) complications (Brownlee, 2005; Volpe et al., 2018; Burgos-Morón et al., 2019). However, the use of antioxidants as therapy isn’t recommended in healthcare practice due to the lack of evidence about their long-term safety and efficacy. Glutathione (GSH) is a major endogenous antioxidant in all cells and determines their redox status and is significantly low in T2D individuals (Townsend et al., 2003). Therefore, replenishing GSH should be a good strategy to improve systemic redox status. However, few clinical trials with GSH supplementation have been conducted in healthy and diabetic individuals. Most of these studies have concentrated on the effect of GSH supplementation on replenishing body stores of GSH; few have studied its impact on reducing oxidative stress, and even fewer on glycemic stress. Results of these trials (Allen and Bradley, 2011; Sekhar et al., 2011; Ritchie et al., 2015) have been difficult to interpret due to differences in the dose and duration of GSH supplementation and the site of outcome measurements, making the clinical recommendations difficult.

Our recent work (Kalamkar et al., 2022) has provided the most conclusive evidence regarding the effects of GSH supplementation in conjunction with antidiabetic treatment. The evidence from this clinical trial suggested that the long-term GSH supplementation offered protection from oxidative damage and improved HbA1c and fasting insulin, especially in elderly T2D patients. We, therefore, believe that GSH should be used as an adjunct therapy for T2D individuals. In our data, we observed significant differences in how individuals respond to GSH intervention. In addition to the factors such as age, diet, physical activity, dose, and length of GSH intervention, the basal amount of endogenous GSH is also responsible for this differential response among individuals. Therefore, we feel that the personalization of GSH supplementation based on endogenous GSH for T2D individuals could be an important addition to current clinical practices. To formulate effective personalized interventions of GSH with antidiabetic treatment, it is essential to understand the dynamics of longitudinal biochemical change and the variations between individual responses to GSH supplementation in detail. This would be largely useful in evaluating the progress of treatment and understanding the glucose control targets for diabetic individuals.

In this work, we have formulated longitudinal mixed-effects models (Laird and Ware, 1982; Brown and Prescott, 2006) to analyze the clinical data of diabetic individuals. Our mixed-effects (ME) models are hierarchical models, where the units of analysis are subject-level predictors (level two) with fixed and random effects. The framework of LME models also performs ‘shrinkage’ for estimating model parameters; that is, individual estimates obtained from LME models are shrunk towards a grand mean of the population level estimate compared to fitting separate linear models to each subject’s data (Bell et al., 2019). ME models have a long history of use in health and medicine since these models treat each patient not only as a member of a population but as an individual with unique characteristics (Gelman et al., 2012; Barr et al., 2013; Baldwin et al., 2014; Wang et al., 2019; Schober and Vetter, 2021). ME models thus allow estimating model parameters that describe between- and within-subject variability of individual responses. A two-level LME model provides reliable estimates in absolute, not just relative, physical units of the variables. This is beneficial for direct clinical use rather than the effect-size-based estimates of treatment effects obtained in our earlier work. We formulated two different LME models, namely, 1) with random intercepts and fixed slopes and 2) random intercepts and random slopes for each variable. These models were evaluated using best likelihood by Akaike’s Information Criteria (AIC) and non-singularity criteria and selected for optimal performance (Bates D. M. et al., 2015).

In our earlier study, we pointed out that the response in elder and younger cohorts was markedly different. We, therefore, analyzed these data separately with LME models.

## Materials and methods

### Clinical trial data

This study has been carried out using the data published in our work (Kalamkar et al., 2022), which was collected from the clinical trial entitled “Effect of glutathione supplementation on glucose homeostasis in diabetic patients” and registered with the Clinical Trials Registry -India (CTRI/2018/01/011257). The data set is freely available online (on the link: https://figshare.com/s/0803267e1d38c054cee6). The analysis of the clinical trial data was conducted with ethical approvals from the Institutional Ethical Committee (IEC) of Jehangir Hospital Development Center, Pune (JCDC ECN- ECR/352/Inst/NIH/2013), IEC of IISER Pune (IECHR/Admin/2019/001); and the Institutional Biosafety Committee (IBC) of SPPU (Bot/27A/15).

The dataset published in the trial comprised 250 known Indian diabetic individuals recruited between February 2016 and January 2018 who were already on anti-diabetic treatment. The clinical trial consisted of three groups: A control group comprising healthy, non-diabetic subjects and two groups of diabetic patients; in one of those, GSH supplementation (500 mg/day for 6 months) was carried out, namely, the DG group, and the other group without supplementation, the D group. The only difference between this D and DG group is the intervention, that is, supplementation with GSH. More importantly, D and DG are similar in nearly all respects, and covariate balance at the baseline has already been shown (Kalamkar et al., 2022).

### Measured variables and follow-up visits

Blood samples of each individual were collected at the time of recruitment and three and 6 months post-GSH supplementation. The dataset used in this study consists of the amounts of reduced (GSH) and oxidized (GSSG) glutathione, fasting and postprandial glucose (FPG and PPG), fasting and postprandial insulin (FPI and PPI), HbA1c, and 8-hydroxy-deoxy-guanosine (8-OHdG), a marker of oxidative DNA damage measured from all individuals.

### Statistical analysis

Descriptive statistics with the mean and standard deviation (SD) were used to describe different study groups in terms of metabolic outcomes at baseline and each subsequent follow-up. Biochemical parameters at different visits were compared using two-sample t-tests. The statistical significance of the comparisons was set at a *p*-value less than 0.05.

### Formulation of linear mixed-effect models

The formulation of linear mixed-effect (LME) models for each biochemical variable (GSH, GSSG, HbA1c, 8-OHdG, FPG, FPI, PPG, and PPI) assumed fixed and random effect parameters at different levels (Level 1: time, Level 2: individuals) in the study. The composite form of the model was written by combining the model equations from these different levels. This form of the model was further used to study the dependency of each effect at different levels and their nested structure in one another. The response variable $Y_{ij}$ from subject *i* on the $j^{th}$ visit was modeled with subject-specific intercepts ( $b_{i0}$ ) and subject-specific slopes ( $b_{i1}$ ) against treatment time $t_{ij}$ (where $t_{ij}$ = 0, 3, 6 months for *j* = 1, 2, 3 visits respectively). An indicator variable $T_{i}$ was assumed to take a value of 0 for the D group and one for the DG group (control and treatment with GSH supplementation, respectively). We denote the average intercept of diabetic individuals when all predictors are 0 by $\beta_{0}$ (mean expected value of the response variable *Y*). $\beta_{1}$ represents the average rate of change in Y during the treatment for the D group. $\beta_{1}+\beta_{2}$ represnts the average rate of change in the DG group. The difference in the rates of change between D and DG $\beta_{2}$ represents the average treatment effect of GSH supplementation on Y.

We considered two candidate models of biochemical variables, namely, 1) random intercept and random slope (RIRS) model and 2) random intercept and fixed slope (RIFS) model for explaining the measured longitudinal data. We formulated RIRS models for the outcome variable $Y_{ij}$ as $Y_{ij}$ = $b_{i0}$ + $b_{i1}$ × $t_{ij}$ + $\epsilon_{ij}$ with subject-specific random slopes and intercepts $b_{i0}$ and $b_{i1}$ defined by $b_{i0}$ = $\beta_{0}$ + $b_{i0}$ and $b_{i1}$ = $\beta_{1}$ + $\beta_{2}$ × $T_{i}$ + $b_{i1}$ where $b_{i0}$ , and $b_{i1}$ were assumed to be distributed as N (0; ${\sigma_{0}}^{2}$ ) and N (0, ${\sigma_{1}}^{2}$ ), with covariance $\sigma_{01}$ , respectively. In the RIRS model, fixed effects are $\beta_{0}$ , $\beta_{1}$ , $\beta_{2}$ and random effects are $b_{i0}$ , $b_{i1}$ . The residual errors were assumed to be normally distributed with a variance of ${\sigma_{e}}^{2}$ . The composite form of the RIRS model for $Y_{ij}$ is given by, $Y_{ij}$ = $\beta_{0}$ + $b_{i0}$ + ( $\beta_{1}$ + $\beta_{2}$ × $T_{i}$ + $b_{i1}$ ) × $t_{ij}$ + $\epsilon_{ij}$ .

RIFS models for outcome variable $Y_{ij}$ were formulated with random intercepts and fixed slopes at subject level (level 2) defined by intercept, $b_{i0}$ = $\beta_{0}$ + $b_{i0}$ and slope; $b_{i1}$ = $\beta_{1}$ + $\beta_{2}$ × $T_{i}$ . The random intercepts $b_{i0}$ in the model were assumed to be distributed as $b_{i0}$ ∼ N (0; ${\sigma_{0}}^{2}$ ). The composite forms of the RIFS model for $Y_{ij}$ is given by $Y_{ij}$ = $\beta_{0}$ + $b_{i0}$ + ( $\beta_{1}$ + $\beta_{2}$ × $T_{i}$ ) × $t_{ij}$ + $\epsilon_{ij}$ .

The design matrices for model equations and covariance matrices are described in further detail in Supplementary Sections S1.1, S1.2.

### Model parameters and fitting

The formulated models have been tested and fitted using the **lme4** package in R (Bates D. et al., 2015); these calculations were confirmed using the **fitlme** package in Matlab and the **mimosa** package (Titz, 2020) for mixed effects models. Other packages, ggplot2, and tidyverse in R, were used for analysis and plots. RIFS and RIRS models were fitted for GSH, GSSG, 8-OHdG, HbA1c, FPG, FPI, PPG, and PPI. A suitable RIFS or RIRS model was selected for each response variable using the best AIC and non-singularity criteria (Bates D. M. et al., 2015).

RIFS models were fitted for five parameters, $\beta_{0}$ , $\beta_{1}$ , $\beta_{2}$ , $\sigma_{0}$ , $\sigma_{e}$ and RIRS models were fitted with seven parameters, $\beta_{0}$ , $\beta_{1}$ , $\beta_{2}$ , $\sigma_{0}$ , $\sigma_{1}$ , $\sigma_{01}$ , $\sigma_{e}$ . The fitted estimates for β and b, the vectors of fixed effect parameters, random effect parameters, respectively, are given by the Best Linear Unbiased Estimator (BLUE) of $\hat{\beta }$ , and Best Linear Unbiased Predictor (BLUP) of $\hat{b}$ , (Refer to Supplementary Section S1.3 for further details). The components of $\hat{b}$ , $b_{i0}$ , and $b_{i1}$ , random effects represent person-specific intercepts (in both RIFS and RIRS) at the baseline and person-specific differences in the rate of change in the slopes (in RIRS only), respectively.

The statistical significance of the results of the LME estimates was determined as *p* < 0.05. We have followed the uncorrected *p*-value to interpret the results through. To ensure completeness, we have performed corrections for multiple comparisons using the Bonferroni method. We applied these corrections for the estimates from LME models for each variable and across all results in both main and supplementary analyses. Those results, which continued to be statistically significant even after the corrections, were marked with a “**#**” in the corresponding tables. The reader should take this into consideration when evaluating the statistical findings.

### Analysis of elder and younger patients

The variation in response to GSH supplementation with age was studied as follows: The data was divided into 1) a subgroup of elder adults (EA) above 55 years and 2) the subgroup of younger adults (YA) below 55 years.

The model for EA is given by $Y_{ij}$ = $\beta_{0}$ + $b_{i0}$ + ( $\beta_{1}$ + $\beta_{2}$ × $T_{i}$ ) × $t_{ij}$ + $\epsilon_{ij}$ . The treatment variable $T_{i}$ takes the value of 0 for EA in the D group and one for the EA in the DG group. The model was formulated similarly for YA as well.

### Analysing the age effects on outcomes

We studied the effects of the age of individuals on the outcome variables Y with different LME models by incorporating 1) continuous variable for the age of individuals at the recruitment and 2) categorical variable for elder and younger age groups. These model formulations are described in Supplementary Section S1.4.

The models considered in this analysis are the following:

(i) Model 1: The original RIRS model in the study without age variables
(ii) Model 2: RIRS model with a treatment-time interaction term, and three-way interaction term with age, treatment indicator, and time at the patient level (Level 2)
(iii) Model 3: RIRS model with a three-way interaction term with age, treatment indicator, and time at the patient level (Level 2)
(iv) Model 4: RIRS model with age groups as a categorical variable for pooling EA and YA at the patient level (Level 2)

These models were fitted for all eight variables, and their performances were compared using AIC and BIC estimates after the likelihood ratio test.

### The structure of the data from the D and DG groups

A sample structure of the data from the clinical trial is given in Supplementary Table S1. This data format was prepared for analysis using the lme4 package. The dataset consisted of eight different measured variables of 201 individuals (100 in D, 101 in DG) who completed both the follow-up visits (3 and 6 months post-GSH supplementation). The Group IDs are encoded as 0 for D and one for DG.

### Estimating correlations between longitudinal changes in different variables

The correlation between individual-specific slopes of variables obtained from RIRS models was estimated using the Pearson correlation coefficient (Pearson, 1895). Correlation diagrams were obtained between all variables using the slopes for RIRS models fitted with 1) the whole data sets and 2) the unpooled data sets from elder individuals and younger individuals. The size of the circle in each cell of the correlation diagram represents the extent of correlation between compared variables. The blue color represents a positive correlation, and the brown represents a negative correlation.

### Making predictions for virtual individuals

The fitted model estimates were utilized to predict responses in virtual individuals with diabetes. We considered three new virtual individuals (V1, V2, and V3) and assumed arbitrary but reasonable baseline measurements of GSH, 8-OHdG, and HbA1c. We thus predicted trajectories in these subjects over 6 months. The scheme used for this purpose is described in Supplementary Section S1.5. The steps in this scheme perform the following:

(i) The baseline values assumed for virtual subjects are shrunk towards the average intercept estimated by our LME model, and the individual specific random effects are obtained.
(ii) Using the LME model estimates of the average intercept, random effect of the intercept, and the rate of changes in the slopes, we obtained the average linear trajectory for each virtual individual in the presence and absence of GSH supplementation.

## Results

### Observational summary of longitudinal changes in the D and DG groups

Group-wise statistics (mean and standard deviation) of the measured variables (GSH, GSSG, 8-OHdG, HbA1c, FPG, FPI, PPG, and PPI) for both D and DG in each of the three visits are described in Kalamkar et al. (2022); these are summarized here for completeness in Table 1.

**TABLE 1:** 0−, 3− and 6− month changes of subjects in D and DG groups. Group-wise means and standard deviations (SD) of blood concentrations of GSH, GSSG, 8-OHdG, HbA1c, FPG, FPI, PPG, and PPI are shown for D and DG groups at different visits. The significance of change is determined for the second (3 months from the first visit) and third visits (6 months from the first visit) relative to the first visit using two-sample t-tests. The significance levels used are ^∗^ *p* < 0.05, ^∗∗^ *p* < 0.01, and ^∗∗∗^ *p* < 0.001. Abbreviations of the variables used here are: HbA1c—glycated hemoglobin, GSH—reduced glutathione, GSSG—oxidized glutathione, PP glucose—postprandial glucose, PP insulin—postprandial insulin, and 8-OHdG–8-hydroxy-2-deoxy guanosine.

Variable	Mean (SD) in the D group			Mean (SD) in the DG group		
	Baseline visit	Second visit	Third visit	Baseline visit	Second visit	Third visit
GSH (μM)	395 (225)	428 (263)	484 (255)^∗∗∗^	465 (352)	1,129 (668)^∗∗∗^	1,021 (518)^∗∗∗^
GSSG (μM)	249 (150)	236 (157)	262 (137)	163 (104)	333 (214)^∗∗∗^	286 (204)^∗∗∗^
8-OHdG (ng/μg DNA)	422 (124)	404 (124)	443 (110)	471 (83)	387 (112)^∗∗∗^	313 (135)^∗∗∗^
HbA1c (%)	8.4 (1.9)	7.9 (1.7)^∗∗^	8.2 (1.8)	8.5 (1.9)	7.7 (1.5)^∗∗∗^	7.9 (1.5)^∗∗∗^
FPG (mg/dL)	160 (61)	143 (47)^∗^	151 (58)^∗^	153 (59)	141 (47)	150 (59)
FPI (μU/mL)	14.2 (10.4)	12.7 (6.8)	12.1 (7.7)	12.6 (8.06)	14.6 (13.8)	13.9 (10.5)^∗∗∗^
PPG (mg/dL)	233.6 (84.1)	216.9 (70.9)	220.3 (83.6)	221.9 (77)	211 (80.4)	218 (83.2)
PPI (μU/mL)	43.4 (26.9)	47.03 (33.3)	40.5 (29.9)	48.3 (47.7)	49.5 (39.6)	52.3 (43.8)

GSH and GSSG were significantly increased, and 8-OHdG and HbA1c significantly decreased (*p* < 0.001) within 3 months in DG and continued to be so at 6 months as well. FPI of DG increased significantly within 6 months (*p* < 0.001). FPG, PPG, and PPI didn’t show significant changes. GSH in the third visit was also significantly increased in D, but not as much compared to the corresponding change in DG.

### LME estimates of the rates of change for the whole population

**FIGURE 1:**
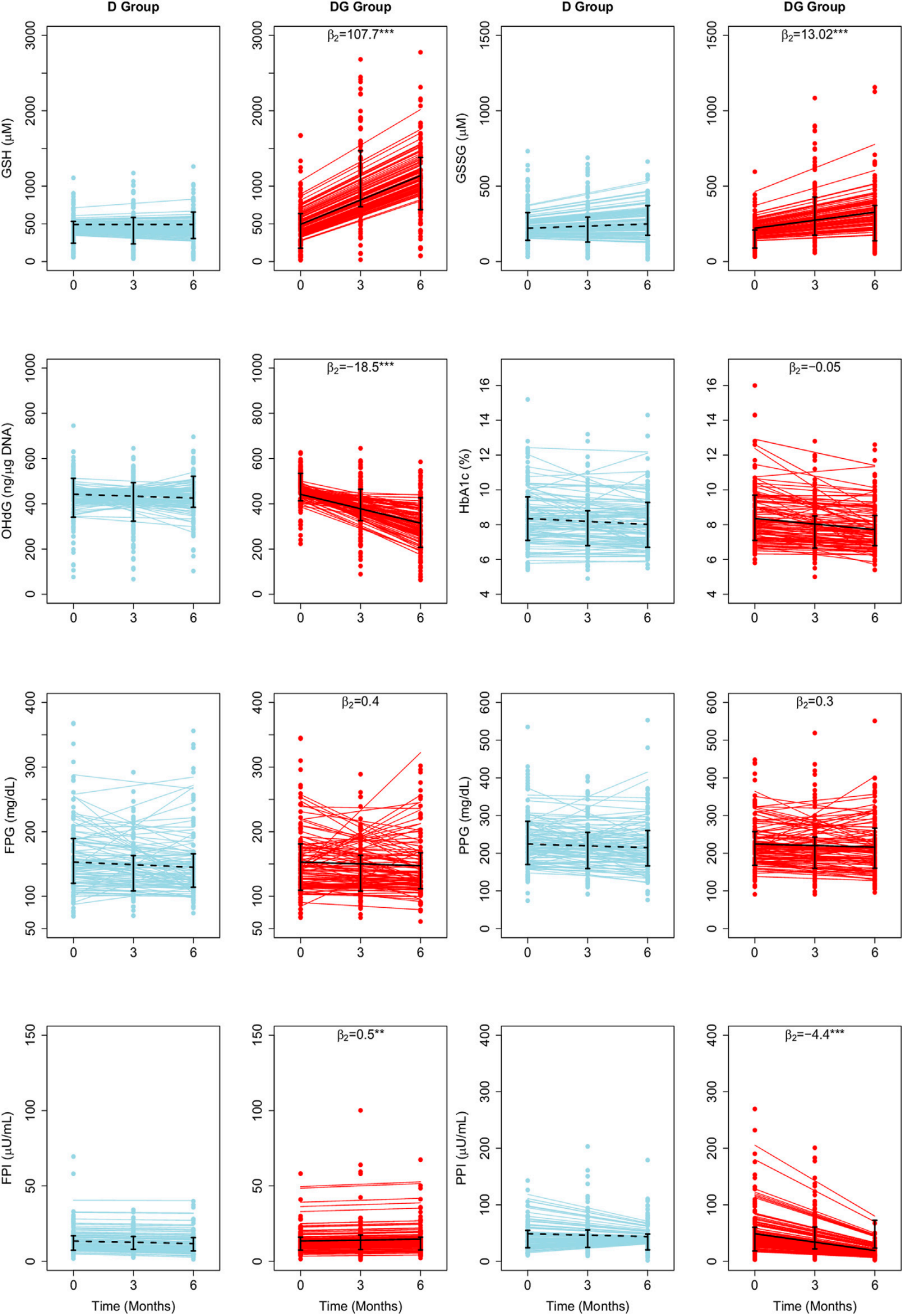
Average treatment effects of GSH supplementation on biochemical changes estimated using LME Models. The fitted results of RIRS models for GSH, GSSG, 8-OHdG, HbA1c, FPG, FPI, PPG, and PPI (RIFS model fits are shown in Supplementary Figure S1) in D group and DG groups (figure panels marked with titles D and DG) are overlaid here with the longitudinal data from 201 individuals (100 D subjects in blue circles, 101 DG subjects in red circles) at different visits. Solid blue and red lines depict the fitted subject-specific mean trajectories in the D group and the DG group, respectively. The black dotted and solid lines represent the group-wise means for D and DG, respectively. Interquartile ranges of the data for D and DG groups are shown with vertical interval plots (25th-75th quartiles) at each visit. The average treatment effects of GSH supplementation ( $\beta_{2}$ ) are denoted on top of each panel corresponding to the DG group. The estimated $\beta_{2}$ was significant on the rate of changes in GSH ( $\beta_{2}$ = 107.7 µM per month), GSSG ( $\beta_{2}$ = 13.02 µM per month), 8-OHdG ( $\beta_{2}$ = −18.5 ng/μg DNA per month), FPI ( $\beta_{2}$ = 0.5 µU/mL per month) and PPI ( $\beta_{2}$ = −4.1 µU/mL per month) levels. The significance levels of parameter estimate are given by ^∗^ *p* < 0.05, ^∗∗^ *p* < 0.01, and ^∗∗∗^ *p* < 0.001. Abbreviations of the variables used here are HbA1c—glycated hemoglobin, GSH—reduced glutathione, GSSG—oxidized glutathione, PP glucose—postprandial glucose, PP insulin—postprandial insulin, and 8-OHdG—8-hydroxy-2-deoxy guanosine.

We fit RIRS and RIFS models for GSH, GSSG, 8-OHdG, HbA1c, FPG, PPG, FPI, and PPI (as described in **Model parameters and fitting**). These subject-wise trajectories obtained from RIRS models are shown in Figure 1. Individual trajectories are distributed around the group-wise average trajectory. Group-wise average intercepts are determined by $\beta_{0}$ ; these are equal for both D and DG. The average slopes in D and DG are $\beta_{1}$ and $\beta_{1}$ + $\beta_{2}$ , respectively. This $\beta_{2}$ denotes the difference between the average slopes in the two groups, that is, the treatment effect of GSH supplementation on outcomes. These estimates ( $\beta_{0}$ ; $\beta_{1}$ , and $\beta_{2}$ ) are detailed in Table 2. Estimated random effects, that is, within-individual and between-individual variations, are described in Supplementary Tables S2, S3.

**TABLE 2:** Fixed-effects parameter values obtained by fitting LME models of RIRS form for GSH, GSSG, 8-OHdG, HbA1c, FPG, FPI, PPG, and PPI variables are presented here with standard error associated with the estimates. Random-effects parameter values are given in Supplementary Table S2. The fitted results from the corresponding RIFS model are shown in Supplementary Table S3. Average treatment effects ( $\beta_{2}$ ) of GSH supplementation were observed to be significant on the rate of changes (slopes) for GSH, GSSG, 8-OHdG, FPI, and PPI levels. Statistical significance levels of parameter estimates are given by ^∗^ *p* < 0.05, ^∗∗^ *p* < 0.01, and ^∗∗∗^ *p* < 0.001. Abbreviations of the variables used here are the same as in Table 1.

Variable	Fixed effect parameters		
	$\beta_{0}$ (SE)	$\beta_{1}$ (SE)	$\beta_{2}$ (SE)
GSH (μM)	492.2 (27.4)^∗∗∗^#	0.04 (8.6)	107.8 (10.3)^∗∗∗^#
GSSG (μM)	221 (11.3)^∗∗∗^#	4.9 (3.1)	12.7 (3.8)^∗∗∗^#
8-OHdG (ng/μg DNA)	442 (7.5)^∗∗∗^#	−2.8 (2.6)	−18.5 (2.9)^∗∗∗^ #
HbA1c (%)	8.4 (0.1)^∗∗∗^ #	−0.06 (0.03)	−0.05 (0.04)
FPG (mg/dL)	152.9 (3.9)^∗∗∗^#	−1.33 (1.09)	0.4 (1.3)
FPI (μU/mL)	13.4 (0.66)^∗∗∗^#	−0.3 (0.14)^∗^	0.5 (0.2)^∗∗^
PPG (mg/dL)	224.4 (5.4)^∗∗∗^#	−1.6 (1.6)	0.3 (1.9)
PPI (μU/mL)	48.8 (2.3)^∗∗∗^#	−0.7 (0.6)	−4.4 (0.7)^∗∗∗^#

We find that $\beta_{2}$ is significant for GSH, GSSG, and 8-OHdG (Table 2). Among the glycemic variables, $\beta_{2}$ is significant only for FPI, and PPI but not for HbA1c, FPG, and PPG.

The mean erythrocytic GSH is estimated as 492 µM in individuals with diabetes. It increased slightly, at an average rate of 0.04 µM per month from the baseline during the study period in D. In DG, GSH increased at an average rate of 107.7 µM per month. Therefore GSH supplementation significantly improved GSH by about 22 percent (107.7 µM, *p* < 0.001) per month relative to baseline. Mean GSSG is estimated as 221 µM. In D and DG, GSSG increased at average rates of 4.7 and 17.7 µM per month, respectively, from the baseline (Figure 1). Thus GSSG rates are significantly improved (*p* < 0.001) by about six percent per month of the baseline (13.02 µM, *p* < 0.001). 8-OHdG is estimated to be 442 ng/μg DNA in diabetic individuals. It decreased in D and DG at average rates of 2.8 and 21.3 ng/μg DNA per month, respectively. Thus the effect of GSH supplementation significantly reduced 8-OHdG by four percent per month of the baseline (18.5 ng/μg DNA, *p* < 0.001).

HbA1c, FPG, and PPG changed at similar rates in D and DG (Figure 1), suggesting that the effect was negligible (*p* > 0.05). FPI and PPI are found to be affected significantly. Mean FPI is estimated as 13.4 µU/mL. FPI decreased at an average rate of 0.3 µU/mL per month in D. GSH supplementation significantly improved FPI at a rate of 0.2 µU/mL in DG. The average PPI is estimated as 48.8 µU/mL in individuals with diabetes. It decreased at average rates of 0.8 and 4.9 µU/mL per month in D and DG, respectively (Figure 1). GSH supplementation significantly enhanced FPI by four percent (0.5 µU/mL, *p* < 0.001) and reduced PPI rates by eight percent (4.1 µU/mL, *p* < 0.001) of the baseline per month.

Results obtained from RIFS models are shown in Supplementary Figure S1 and Supplementary Table S3. The parameter estimates of $\beta_{2}$ from RIFS models are also found to be significant for GSH, GSSG, 8-OHdG, FPI, and PPI, leading to similar conclusions about the effects of GSH supplementation as in RIRS models.

We note that these results largely coincide with the results from previous work (Kalamkar et al., 2022). However, FPI and PPI, which were earlier reported not to be affected by GSH supplementation, are found to have a significant effect through the LME model-based analysis.

### Independent LME model estimates for ages above and below 55 years

Diabetes is an age-onset disease; an early diagnosis leads to an increased chance for complications to set in relatively early. We have earlier demonstrated that the effectiveness of GSH supplementation differed between the younger and elder populations using an age cutoff of 55 years, which was the median age of the study population (Kalamkar et al., 2022). We fit a separate LME for each of these two age groups. Model estimates obtained by fitting LME models independently for EA and YA are detailed in Supplementary Table S4.

GSH supplementation significantly affected GSH, 8-OHdG, HbA1c, FPI, and PPI in EA, and GSH, GSSG, 8-OHdG, and PPI in YA ( $\beta_{2}$ in Table 3, *p* < 0.001).

**TABLE 3:** Baseline assumptions for virtual individuals. The concentrations of GSH, 8-OHdG, and HbA1c assumed at the baseline for virtual individuals (V1, V2, and V3) to make predictions using RIFS models are shown in the table.

Subject ID	GSH (μM)	8-OHdG (ng/μg DNA)	HbA1c (%)
V1	200	500	10
V2	500	400	8
V3	800	300	6

#### GSH

**FIGURE 2:**
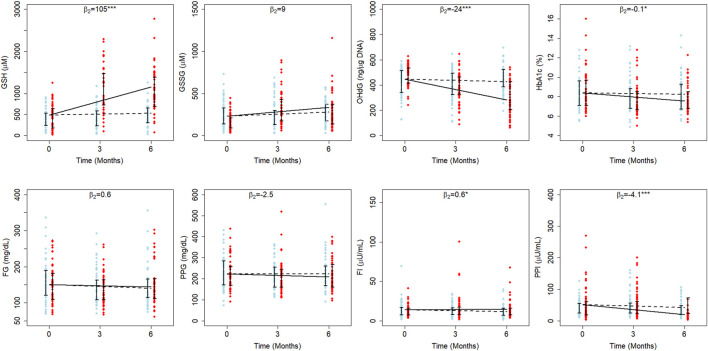
Average treatment effects of GSH supplementation in elder diabetics. The fitted results of RIRS models for GSH, GSSG, 8-OHdG, HbA1c, FPG, FPI, PPG, and PPI variables (RIFS model fits are shown in Supplementary Figure S4) of elder adults (EA) are shown on different panels here with the longitudinal data (blue circles for D individuals and red circles for DG individuals) at different visits. The data from 107 elder adults (52 from D and 55 from DG) are overlaid with group-wise mean trajectories for D and DG groups represented by black dotted lines and solid lines, respectively. Interquartile data ranges for individuals (from D and DG) are shown with vertical interval plots (25th-75th quartiles) at each visit. The average treatment effects of GSH supplementation ( $\beta_{2}$ ) on the rate of changes (slope) denoted on top of corresponding panels which are significant on GSH ( $\beta_{2}$ = 104 µM per month), 8-OHdG ( $\beta_{2}$ = −23.7 ng/μg DNA per month), HbA1c ( $\beta_{2}$ = −0.1% per month), FPI ( $\beta_{2}$ = 0.6 µU/mL per month), and PPI ( $\beta_{2}$ = −3.6 µU/mL per month) in elder adults. The significance of these parameter estimates and abbreviations of the variables are the same as in Figure 1.

Mean erythrocytic GSH in EA (488 µM) is estimated to be less than YA (497 µM). In YA of D, it decreased at an average rate of 6.9 µM per month, whereas in DG, GSH increased at an average rate of 104 µM per month (Supplementary Figure S2). In EA of D and DG, GSH increased at average rates of 6.5 and 111 µM per month, respectively (Figure 2). This clearly indicates that GSH supplementation resulted in a significant improvement in GSH by about 21 percent per month of their baseline in YA (111 μM, *p* < 0.001) and 22 percent per month in EA (105 μM, *p* < 0.001) with diabetes.

#### GSSG

Interestingly, the effect on GSSG was significant in YA (*p* < 0.01) but not in EA. The mean GSSG in EA (231 µM) was estimated to be higher than YA (209 µM). When YA of D and DG were examined, GSSG increased at average rates of 1.9 and 18.4 µM per month, respectively (Supplementary Figure S2). It increased at average rates of 7.6 and 17.1 µM per month in EA of D and DG, respectively (Figure 2). This shows that GSH supplementation enhanced GSSG significantly per month by eight percent of the baseline (17.5 µM, *p* < 0.001) per month only in YA.

#### 8-OHdG

The average 8-OHdG estimate is higher in EA (445 ng/μg DNA) than in YA (438 ng/μg DNA). In EA of both D and DG, 8-OHdG decreased at average rates of 3.3 and 27 ng/μg DNA per month during the study period (Figure 2). Similarly, it decreased at average rates of 2.1 and 14.16 ng/μg DNA per month in the YA of D and DG groups (Supplementary Figure S2). Thus, we find that GSH supplementation significantly reduced 8-OHdG from the baseline by 12.06 ng/μg DNA per month (3%) in YA and 23.7 ng/μg DNA per month (5%) in EA. These results suggest that oral GSH administration rapidly offers better protection from oxidative DNA damage in EA compared to YA.

#### HbA1c

GSH supplementation was earlier reported to affect the HbA1c in the elder cohort significantly (Kalamkar et al., 2022). We examined LME estimates of both YA and EA to quantitate the effect on HbA1c. The average HbA1c is estimated at 8.3% and 8.4% in YA and EA, respectively. In EA of D, HbA1c decreased at an average rate of 0.02% per month, while in DG, it decreased at an average rate of 0.12% per month (Figure 2), suggesting that GSH supplementation improved HbA1c rates significantly by about 0.1% per month in EA. Estimated HbA1c rates are not significantly different between YA of D and DG (Supplementary Figure S2).

#### Fasting Insulin

Our earlier work (Kalamkar et al., 2022) found that oral GSH supplementation significantly changed FPI in elder patients. We quantitated the effect on FPI using LME model estimates (Supplementary Table S4). The average FPI is estimated to be 12.9 µU/mL in YA and 14 µU/mL in EA. In both EA and YA of D, FPI decreased at rates of 0.4 µU/mL and 0.1 µU/mL per month, respectively (Figure 2). The estimated rates were similar between the YA of the D and DG, indicating that the effect on FPI is negligible (*p* > 0.05). On the other hand, in EA of DG, FPI increased at a rate of 0.2 µU/mL per month, suggesting that GSH supplementation improved FPI rates significantly by 0.6 µU/mL per month. FPI increased by 4.3% of the baseline per month in EA and negligibly in YA.

#### Postprandial Insulin

Using LME models to fit the data, PPI was found to decrease in both YA and EA. The average PPI in YA and EA is estimated to be 46 and 51 µU/mL, respectively. In YA of D, PPI increased at a rate of 0.1 µU/mL per month, whereas in DG, it decreased at a rate of 4.7 µU/mL per month. PPI decreased at average rates of 1.6 µU/mL and 5.2 µU/mL per month in EA of D and DG, respectively.

#### Fasting and Postprandial Glucose

The average FPG estimated in YA and EA are 156 and 150 mg/dL, respectively. In both YA and EA, the GSH supplementation effect wasn’t found to be significant. In both EAs of D and DG, FPG decreased at average rates of 1.7 and 0.9 mg/dL per month, respectively. Similarly, in YAs of D and DG, it decreased at average rates of 1.3 and 0.8 mg/dL per month, respectively. PPG estimated in YA and EA at the time of recruitment is 227 and 223 mg/dL, respectively. GSH supplementation decreased PPG by 2.5 mg/dL per month in EAs and increased PPG by 3.5 mg/dL per month in YA.

For exploratory purposes, we also analyzed the effects of the age using new candidate models as incorporated with age as a model variable (Model 2, Model 3, and Model four in Supplementary Section S1.4) for GSH, GSSG, 8-OHdG, HbA1c, FPG, FPI, PPG, and PPI. Results obtained by fitting with these models are shown in Supplementary Tables S5A–C. When we compared model fits from all four models using AIC and BIC estimates, our original RIRS model (Model 1) was found to be the better-fit model for all variables (Supplementary Table S5D).

### Changes in GSH correlate strongly with changes in HbA1c and 8-OHdG in EA

**FIGURE 3:**
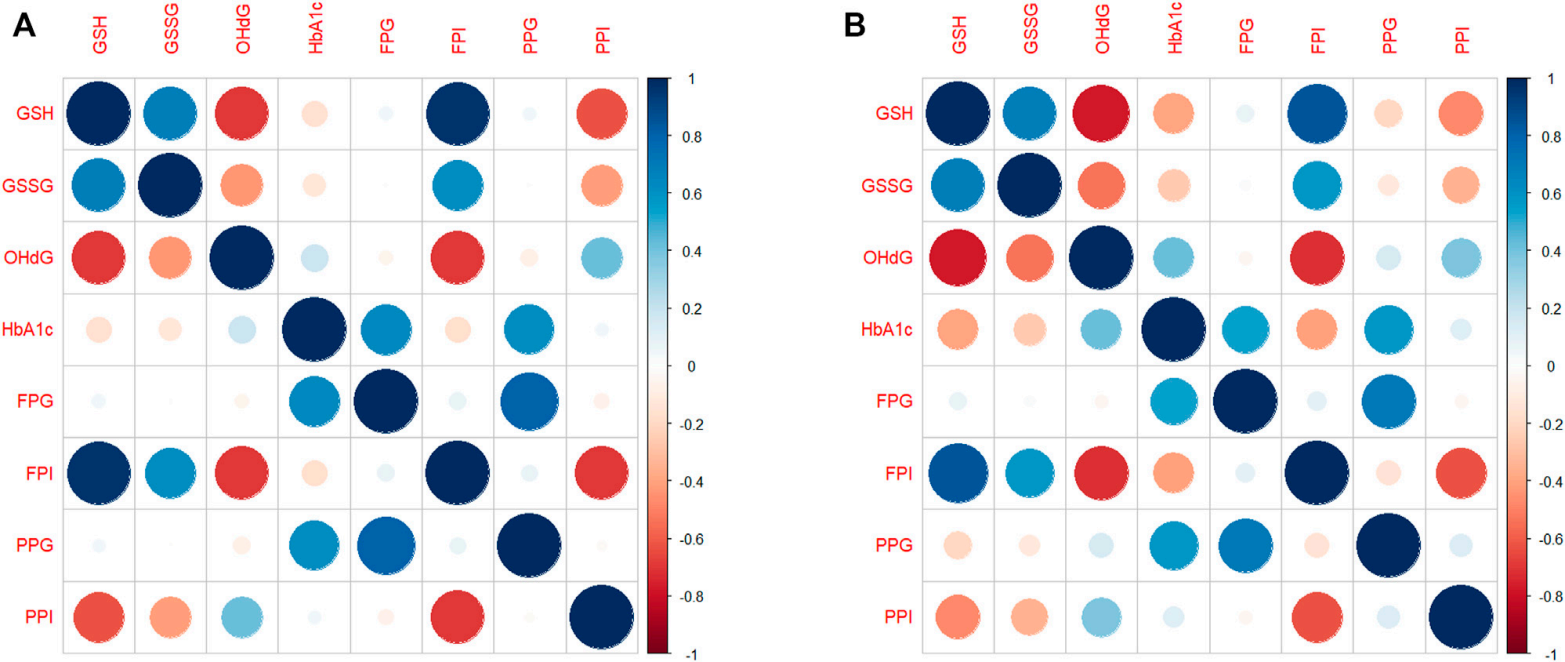
Correlation diagram between subject-specific changes **(A)** for the whole population and **(B)** for EAs. The correlation diagrams obtained between subject-specific random slopes from fitted RIRS models for different biochemical measures (GSH, GSSG, 8-OHdG, HbA1c, FPG, FPI, PPG, and PPI) are shown here. The strength and direction of correlation between subject-specific slopes are reflected in both color and size of the circular markers. The scales of Pearson’s correlation coefficient have been classified as low (r < 0.4), moderate (r < 0.6), strong (r > 0.6), or very strong (r > 0.8). Blue indicates a strong positive correlation, and red indicates a strong negative correlation. Abbreviations of the variables are the same as in Figure 1.

We estimated pairwise correlations between subject-specific slopes of GSH, GSSG, 8-OHdG, HbA1c, FPG, FPI, PPG, and PPI obtained from RIRS models. These correlation diagrams for the full population (pooled data) are shown in Figure 3A. Changes in GSH are found to be strongly correlated positively with GSSG (r > 0.6) and FPI (r > 0.9). Changes in GSH correlated negatively with 8-OHdG and PPI (r < −0.6). The other correlations are found to be relatively weaker.

Correlation plots for EAs alone are shown in Figure 3B. GSH slopes are strongly negatively correlated with 8-OHdG slopes (r = −0.71) and HbA1c slopes at moderate levels (r = −0.43). GSH slopes are strongly negatively correlated with PPI slopes (r = −0.74, Figure 3B); however, they are strongly positively correlated with FPI (r = 0.75).

In YAs (Supplementary Figure S3), GSH slopes are negatively correlated at moderate levels with 8-OHdG (r = −0.43) and PPI (r = −0.57) slopes. The correlation between GSH slopes and HbA1c slopes is negligibly small.

Taken together, the strengths of the correlations between the changes in GSH and outcome variables are evidently different between EAs and YAs.

We next use LME model estimates to help quantify the overall rates of changes that can be expected of individuals.

### Predicted trajectories for virtual diabetic individuals

Next, we describe the sample predictions obtained for three virtual individuals (V1, V2, and V3) using RIFS models. Baseline values assumed for these virtual individuals are given in Table 3.

**FIGURE 4:**
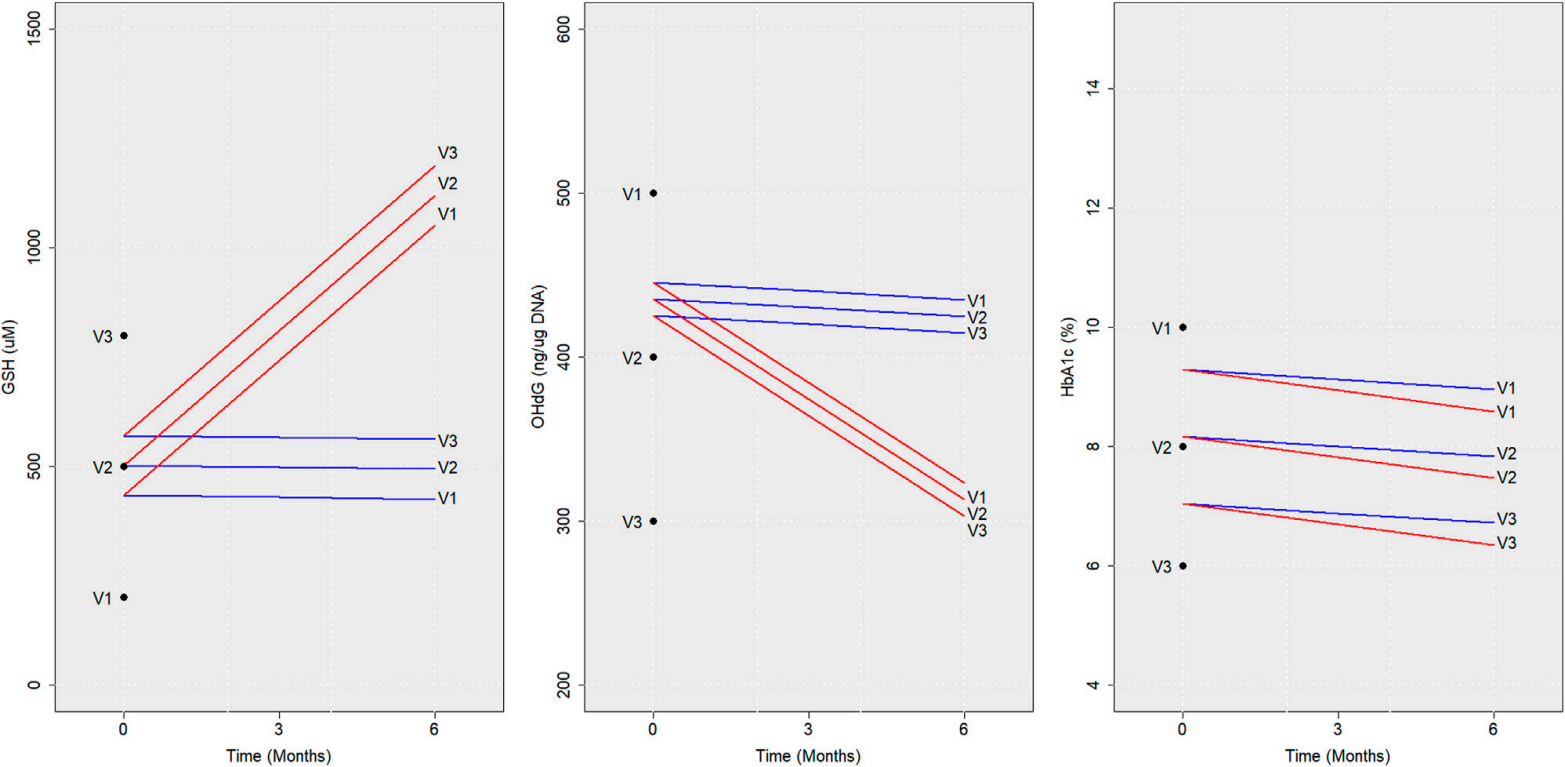
Model predictions for virtual individuals. Average trajectories of the concentration of GSH, 8-OHdG, and HbA1c predicted using RIFS models in virtual individuals (V1, V2, and V3) if they were to be followed up with GSH supplementation (red) and without GSH supplementation (blue) are shown for 6 months are depicted here. The baseline values assumed and the values predicted after 6 months are also marked for V1, V2, and V3. Abbreviations of the variables are the same as in Figure 1.

The trajectories of GSH, 8-OHdG, and HbA1c obtained if they were with or without GSH supplementation are shown in Figure 4. RIFS models predicted the GSH of V1 close to 429 µM by the end of 6 months, whereas, on GSH supplementation, V1 ended up at 1,079 µM. Similar predictions were made for 8-OHdG and HbA1c for all these individuals (Figure 4).

This can also be modified to estimate 1) the average time required for a recruited individual to reach a particular level of a biochemical parameter given the baseline value and 2) the expected change in the level of a particular biochemical parameter with time.

Finding a patient’s potential trajectory has direct clinical and academic uses. This method, therefore, can be used on newly added subjects to predict different outcomes during 6 months, with or without GSH supplementation.

## Discussion

Our earlier study demonstrated *population-level* changes in GSH, GSSG, HbA1c, 8-OHdG, FPG, FPI, PPG, and PPI; these changes were further studied for younger and elder subgroups of the patients. The response in individual patients is, unsurprisingly, considerably varied; however, analyzing individual responses was beyond the scope of that study. In the present study, we are focused on explaining *individual-level* responses to GSH supplementation over the full study period of 6 months. We addressed this through a linear mixed-effects model framework. The major results of this study are to characterize the variability in the inter-individual biochemical response, in particular, determined by the age group of an individual. To the best of our knowledge, this is the first inter-individual analysis of the effects of GSH supplementation in patients with diabetes.

The response to GSH supplementation was analyzed in the earlier work (Kalamkar et al., 2022) by comparing 6-month changes in D and DG groups through population-level Cohen’s-d-based estimates. GSH supplementation was found to significantly affect GSH, GSSG, and 8-OHdG levels (at moderate levels of Cohen’s d > 0.6) and not for HbA1c, FPG, FPI, and PPG variables. The LME model framework helped analyze biochemical responses longitudinally and obtain more refined estimates that account for inter-individual and within-individual variations at two levels of hierarchy. We note that LME models describe linear trajectories over a 6-month duration. The estimates show that D and DG average trajectories lie between the 25th and 75th percentiles of the data at all visits; that is, these models are a good description of the data.

Model estimates were consistent with the effect size estimates in the earlier study (Kalamkar et al., 2022) for GSH, GSSG, 8-OHdG, HbA1c, FPG, FPI, and PPG variables but not for PPI. LME estimates determined that the GSH supplementation markedly enhanced the rate of replenishments in erythrocytic GSH stores by about 22%, GSSG stores by about 6%, and reduced oxidative DNA damage by about 4% of the baseline month in diabetic patients. Importantly, these estimates are in the actual (not relative) physical units and are, therefore, directly interpretable for use in clinical applications.

We had identified an older subgroup separate from a younger diabetic population that benefits better from GSH supplementation through a *post hoc* subgroup analysis in our earlier study. That study wasn’t designed to evaluate this analysis explicitly, and as such, it was a weaker form of evidence. LME models provided a more formal way of comparing their differential responses; that is, two independent models described the responses in each of these two age classes. GSH supplementation improved the rates of 8-OHdG and HbA1c reduction in elder diabetic individuals more than in younger diabetic cohorts. LME models estimated the effect to be significant for FPI in elder patients, which supported our claims of a beneficial elder cohort. Model estimates for GSSG suggested a significant effect of GSH supplementation in younger patients (by 17 µM per month) but not in elder ones. In contrast to the earlier results, PPI model estimates were found to be significant in both elder and younger cohorts. Thus, our model-based analysis describes the extent to which diabetic patients above 55 can be expected to benefit from GSH supplementation.

LME model estimates further allow for examining the strength of the association between covariates. The results of the correlation analysis (in Figure 3; Supplementary Figure S3) show to what extent GSH intervention improves erythrocytic GSH stores and reduces DNA damage. Estimates from the elder and younger individuals also revealed that GSH changes were correlated strongly with changes in HbA1c and 8-OHdG in elder adults.

Finally, we have formulated a scheme (in Supplementary Section S1.5) that makes individual-specific *predictions* for newly recruited subjects with diabetes, given a baseline measurement by using the LME model estimates of the fixed-effects and random-effects parameters. In particular, this scheme can be utilized to make predictions of what changes might be expected in the biochemical levels. Alternatively, the average time required for a recruited patient to reach a particular range of biochemical parameters in diabetic subjects can be estimated. The fitted LME model estimates can be used to identify the extent of each subject’s response, whether they are in a better or worse condition than the average population response (Inzucchi et al., 2012; Kirkman et al., 2012). These schemes are of direct clinical and academic use to predict prospective trajectories, which can be a powerful addition to the clinician’s toolbox.

Strengths of this study include that it is based on the data available from diabetic individuals on a well-conducted, randomized control trial, which is one of the most extensive GSH supplementation studies so far. Using LME models, we evaluated the individual trajectories and associated variations within individuals and between individuals, which has not been done before in GSH intervention studies.

It is particularly important to keep in mind that our understanding of the results is based on the uncorrected *p* values. The practice of correcting for multiple comparisons has been a topic of debate among statisticians for several years now. Various opinions were found in the literature in opposition regarding the conditions under which a correction for multiple testing should be applied. We note that several highly cited reports over the years (Poole, 1991; Perneger, 1998; Cabin and Mitchell, 2000) recommend dismissing the usage of corrections with multiple comparisons. It was shown that when trying to reduce the rate of false positives (Type I error) for null associations, often leads to an increase in the rate of false negatives (Type II error) for those that are not null (Rothman, 1990). Also, these comparisons were often complained of being unnecessarily conservative, which makes this approach frequently fails to identify actual differences. However, for the interest of all readers, we have also incorporated significance levels after corrections for each comparison. Those readers who prefer statistically corrected results should follow the corresponding tables to determine which findings still retain significance and which did not after correction for multiple comparisons.

We had earlier identified the differential effects of GSH supplementation in elder and younger subgroups (Kalamkar et al., 2022). This study analyzed the longitudinal responses of GSH supplementation observed in these subgroups of diabetic individuals rigorously with a framework of the LME models. The subgroup of subjects above the median age of 55 is consistent with previous studies that show an increased risk of diabetes-related complications in individuals around this age. Several organizations have already developed guidelines specific to, or including, older adults on their annual Standards of Medical Care in Diabetes (American Diabetes Association, 2012). These reports also discuss the severity of diabetes complications in elders and the lack of high-level evidence on the effectiveness of different medications in diabetics (Leung et al., 2018). We think the onset of diabetes and complications should be addressed differently for elder and younger diabetic individuals, and treatments need to be planned separately from each other. The two independent LME models formulated for analyzing the longitudinal trajectories of elder and younger adults provided estimates of the treatment effect of GSH supplementation on each endpoint separately. This helps in identifying their extent of recovery and examining whether individuals are in a better or worse condition than the average profile in these subgroups on GSH supplementation for direct clinical use. We recommend planning large-scale clinical trials to examine these insights about GSH supplementation, especially in elder diabetic individuals. This could help in establishing novel benchmarks for caring for elder patients with diabetes. We have also analyzed different possible models to study the effect of the age of individuals on GSH supplementation. This will form the basis and motivate a number of future studies to examine many of the finer nuances of the effect of age on supplementation.

Some limitations of this study also need to be considered. Although antidiabetic treatments were not changed during the period of the study, patients did use different types of medication. We have not analyzed the combinatorial complexity of treatments further due to a lack of sufficient statistical power. It is possible that future work may uncover if GSH supplementation is particularly more effective with certain treatments than others. The results presented here can be the basis for future GSH intervention studies that advance precision diabetes research.

## Data Availability

Publicly available datasets were analyzed in this study. This data can be found here: 10.6084/m9.figshare.21786518.

## Abbreviations

T2D, Type 2 diabetes; HbA1c, glycated hemoglobin; GSH, reduced glutathione; GSSG, oxidized glutathione; PP glucose, postprandial glucose; PP insulin, postprandial insulin; 8-OHdG, 8-hydroxy-2-deoxy guanosine; LME, Linear Mixed-Effects; EA, Elder Adults.
