# Supplementary material for: Pune GSH supplementation study: Analyzing longitudinal changes in type 2 diabetic patients using linear mixed-effects models
Source: Front Pharmacol. 2023 Mar 13;14:1139673. doi: 10.3389/fphar.2023.1139673 (PMC10040593; doi:10.3389/fphar.2023.1139673)
Supplement: Supplementary file 1 [file DataSheet1.PDF]

Pune GSH Supplementation Study: Analyzing longitudinal changes in type 2 diabetic patients using linear mixed-effects models

1 Supplemental Data

1.1 Design matrix of model equations

The matrix version of the equations was used to explain the estimates for all subjects from the mixed model equation given by

$$Y = X\beta + Zb + \epsilon \tag{1}$$

where the data vector (Y) and design matrices for RIRS model (with $N = 201$ subjects for whole group comparisons) used are

$$Y = \begin{pmatrix} Y_{11} \\ Y_{12} \\ Y_{13} \\ Y_{21} \\ Y_{22} \\ Y_{23} \\ .. \\ .. \\ Y_{N1} \\ Y_{N2} \\ Y_{N3} \end{pmatrix} \quad X = \begin{pmatrix} 1 & t_{11} & T_1 \times t_{11} \\ 1 & t_{12} & T_1 \times t_{12} \\ 1 & t_{13} & T_1 \times t_{13} \\ 1 & t_{21} & T_2 \times t_{11} \\ 1 & t_{22} & T_2 \times t_{12} \\ 1 & t_{23} & T_2 \times t_{13} \\ .. & .. & .. \\ .. & .. & .. \\ 1 & t_{N1} & T_N \times t_{11} \\ 1 & t_{N2} & T_N \times t_{12} \\ 1 & t_{N3} & T_N \times t_{13} \end{pmatrix} \quad \beta = \begin{pmatrix} \beta_0 \\ \beta_1 \\ \beta_2 \end{pmatrix} \quad Z = \begin{pmatrix} 1 & t_{11} & 0 & 0 & .. & 0 & 0 \\ 1 & t_{12} & 0 & 0 & .. & 0 & 0 \\ 1 & t_{13} & 0 & 0 & .. & 0 & 0 \\ 0 & 0 & 1 & t_{21} & .. & 0 & 0 \\ 0 & 0 & 1 & t_{22} & .. & 0 & 0 \\ 0 & 0 & 1 & t_{23} & .. & 0 & 0 \\ .. & .. & .. & .. & .. & .. & .. \\ .. & .. & .. & .. & .. & .. & .. \\ .. & .. & .. & .. & .. & .. & .. \\ 0 & 0 & 0 & 0 & .. & 1 & t_{N1} \\ 0 & 0 & 0 & 0 & .. & 1 & t_{N2} \\ 0 & 0 & 0 & 0 & .. & 1 & t_{N3} \end{pmatrix} \quad b = \begin{pmatrix} b_{10} \\ b_{11} \\ b_{20} \\ b_{21} \\ .. \\ .. \\ b_{N0} \\ b_{N1} \end{pmatrix} \quad \epsilon = \begin{pmatrix} \epsilon_{11} \\ \epsilon_{12} \\ \epsilon_{13} \\ \epsilon_{21} \\ \epsilon_{22} \\ .. \\ .. \\ \epsilon_{N1} \\ \epsilon_{N2} \\ \epsilon_{N3} \end{pmatrix}$$

The data vector and design matrices for RIFS model used are

$$Y = \begin{pmatrix} Y_{11} \\ Y_{12} \\ Y_{13} \\ Y_{21} \\ Y_{22} \\ Y_{23} \\ .. \\ .. \\ Y_{N1} \\ Y_{N2} \\ Y_{N3} \end{pmatrix} \quad X = \begin{pmatrix} 1 & t_{11} & T_1 \times t_{11} \\ 1 & t_{12} & T_1 \times t_{12} \\ 1 & t_{13} & T_1 \times t_{13} \\ 1 & t_{21} & T_2 \times t_{11} \\ 1 & t_{22} & T_2 \times t_{12} \\ 1 & t_{23} & T_2 \times t_{13} \\ .. & .. & .. \\ .. & .. & .. \\ 1 & t_{N1} & T_N \times t_{11} \\ 1 & t_{N2} & T_N \times t_{12} \\ 1 & t_{N3} & T_N \times t_{13} \end{pmatrix} \quad \beta = \begin{pmatrix} \beta_0 \\ \beta_1 \\ \beta_2 \end{pmatrix} \quad Z = \begin{pmatrix} 1 & 0 & .. & 0 & 0 \\ 1 & 0 & .. & 0 & 0 \\ 1 & 0 & .. & 0 & 0 \\ 0 & 1 & .. & 0 & 0 \\ 0 & 1 & .. & 0 & 0 \\ 0 & 1 & .. & 0 & 0 \\ .. & .. & .. & .. & .. \\ .. & .. & .. & .. & .. \\ 0 & 0 & 0 & .. & 1 \\ 0 & 0 & 0 & .. & 1 \\ 0 & 0 & 0 & .. & 1 \end{pmatrix} \quad b = \begin{pmatrix} b_{10} \\ b_{20} \\ .. \\ .. \\ b_{N0} \end{pmatrix} \quad \epsilon = \begin{pmatrix} \epsilon_{11} \\ \epsilon_{12} \\ \epsilon_{13} \\ \epsilon_{21} \\ \epsilon_{22} \\ .. \\ .. \\ \epsilon_{N1} \\ \epsilon_{N2} \\ \epsilon_{N3} \end{pmatrix}$$

1.2 Covariance matrices in the model

The covariance matrix for the data vector Y (from N subjects), V, fitted with LME model of the form $Y = X\beta + Zb + \epsilon$, is obtained as $V = ZGZ^T + R$, where the expectation values $\mathbb{E}(b) = 0$, $\mathbb{E}(\epsilon) = 0$, covariance matrix of random effects, $Var(b) = G$, and the covariance matrix for residual error, $Var(\epsilon) = R$. The structure of G in RIRS model is of a block diagonal matrix form

$$G = \begin{pmatrix} \sigma_0^2 & \sigma_{01} & 0 & 0 & .. & .. & 0 & 0 \\ \sigma_{01} & \sigma_1^2 & 0 & 0 & .. & .. & 0 & 0 \\ 0 & 0 & \sigma_0^2 & \sigma_{01} & .. & .. & 0 & 0 \\ 0 & 0 & \sigma_{01} & \sigma_1^2 & .. & .. & 0 & 0 \\ .. & .. & .. & .. & .. & .. & .. & .. \\ .. & .. & .. & .. & .. & .. & .. & .. \\ 0 & 0 & 0 & 0 & .. & .. & \sigma_0^2 & \sigma_{01} \\ 0 & 0 & 0 & 0 & .. & .. & \sigma_{01} & \sigma_1^2 \end{pmatrix}$$

This matrix is composed of N identical blocks $\begin{pmatrix} \sigma_0^2 & \sigma_{01} \\ \sigma_{01} & \sigma_1^2 \end{pmatrix}$ corresponding random effects from N subjects. Similarly, the structure of the covariance matrices for RIFS model looks like

$$G = \begin{pmatrix} \sigma_0^2 & 0 & 0 & .. & .. & 0 \\ 0 & \sigma_0^2 & .. & .. & 0 & 0 \\ .. & .. & .. & .. & .. & .. \\ .. & .. & .. & .. & .. & .. \\ 0 & 0 & 0 & .. & .. & \sigma_0^2 \end{pmatrix}$$

which was composed of N identical blocks of $\left(\sigma_0^2\right)$. The structure of R was assumed to be a diagonal matrix, with $R = \sigma_e^2 \mathbb{I}_{3N\times 3N}$, where $\mathbb{I}_{3N\times 3N}$ is a block diagonal matrix with N identical blocks of identity matrices for N subjects with dimension 3×3 .

1.3 Model parameters and fitting

The formulated models have been tested and fitted using the lme4 package in R, and fitlme package in Matlab. The results of these 2 level models have been verified using Mimosa UI application for mixed effects models. Other packages, ggplot2, and tidyverse in R was used for analysis and plots. The choice between RIFS and RIRS models were made to obtain the best model estimates of the treatment effects based on best AIC values and by avoiding the singularity arising while fitting models (Barr et al. 2013).

The fixed-effects and random-effects parameters are obtained by fitting the model meets singularity criterion and with highest likelihood. RIFS models were fitted for 5 parameters, $\beta_0, \beta_1, \beta_2, \sigma_0, \sigma_e$, and RIRS models were fitted with 7 parameters, $\beta_0, \beta_1, \beta_2, \sigma_0, \sigma_1, \sigma_{01}, \sigma_e$. The fitted estimates for β and b, vectors of fixed effect parameters, random effect parameters respectively are given by the Best Linear Unbiased Estimator (BLUE) of $\hat{\beta}$, and Best Linear Unbiased Predictor (BLUP) of $\hat{b}$

$$\hat{\beta} = (X^T V^{-1} X)^{-1} X^T V^{-1} Y \tag{2}$$

$$\hat{b} = GZ^T V^{-1}(Y - X\beta) \tag{3}$$

The components of $\hat{b}$, b_{i0}, and b_{i1}, random effects represent person-specific intercepts (in both RIFS and RIRS) at the baseline and person-specific differences in the rate of change in the slopes (in RIRS only), respectively.

1.4 LME Models to study age effects

We formulated different LME models for analyzing the age effects on the outcome variables Y. Model 1 below represents the original model formulated in the main manuscript. The other three models (Model 2, Model 3, Model 4) are used to analyze the data, and fitted results are shown in Supplementary Table S5 A-C. The comparison between these models is given in Supplementary Table S5D.

- **Model 1**: (original model)
$$Y_{ij} = b_{i0}^* + b_{i1}^* \times t_{ij} + \epsilon_{ij} \tag{4}$$
where subject-specific random slopes and intercepts b_{i0}^* and b_{i1}^* defined by
$$b_{i0}^* = \beta_0 + b_{i0}$$
$$b_{i1}^* = \beta_1 + \beta_2 \times T_i + b_{i1}$$

- **Model 2**: The outcome (Y) was modeled with a three-way interaction term of age (Age_i), treatment (T_i), and time (t_{ij}) below as:
$$Y_{ij} = b_{i0}^* + b_{i1}^* \times t_{ij} + \epsilon_{ij} \tag{5}$$
where subject-specific random slopes and intercepts b_{i0}^* and b_{i1}^* defined by
$$b_{i0}^* = \beta_0 + b_{i0}$$
$$b_{i1}^* = \beta_1 + \beta_2 \times T_i Age_i + b_{i1}$$

- **Model 3**: The outcome (Y) was modeled with a treatment and time interaction term and a three-way interaction term of age (Age_i), treatment (T_i), and time (t_{ij}) below as:
$$Y_{ij} = b_{i0}^* + b_{i1}^* \times t_{ij} + \epsilon_{ij} \tag{6}$$
where subject-specific random slopes and intercepts b_{i0}^* and b_{i1}^* defined by
$$b_{i0}^* = \beta_0 + b_{i0}$$
$$b_{i1}^* = \beta_1 + \beta_2 \times T_i + \beta_3 \times T_i Age_i + b_{i1}$$

- **Model 4**: The outcome (Y) was modeled with categorical treatment variables for $Age \geq 55$ and $Age < 55$ as
$$Y_{ij} = b_{i0}^* + b_{i1}^* \times t_{ij} + \epsilon_{ij} \tag{7}$$
where subject-specific random slopes and intercepts b_{i0}^* and b_{i1}^* defined by
$$b_{i0}^* = \beta_0 + b_{i0}$$
$$b_{i1}^* = \beta_1 + \beta_2 \times T_i^{Age \geq 55} + \beta_3 \times T_i^{Age<55} + b_{i1}$$

These four models are used to fit GSH, GSSG, 8-OHdG, HbA1c, FPG, FPI, PPG, and PPI data, and the results obtained are given in Supplementary Table S5. This model fits were also compared using AIC and BIC values in Supplementary Table S7.

1.5 Prediction for virtual subjects

We make predictions for the trajectories of new virtual subjects (VS1, VS2, VS3) by assuming their baseline measurements of GSH, 8-OHdG, and HbA1c using the following steps.

1. Obtain the model estimates of fixed-effects, $\hat{\beta} = (\beta_0, \beta_1, \beta_2)$, random effects and covariance parameters σ_0, σ_e.

2. Random-intercepts for new subjects estimated based on the baseline data (Y^{New}) for subjects VS1, VS2, and VS3 as
$$b_i^{New} = GZ^T V^{-1}(Y^{New} - X\hat{\beta}) \tag{8}$$

3. Predict the average trajectories for new subjects using the subject-specific random-effects b^{New} and design matrices, X^{Pred} and Z^{Pred} with fixed-effects and random-effects parameters, respectively, as
$$Y^{Pred} = X^{Pred}\beta + Z^{Pred}b^{New} \tag{9}$$

2 Supplementary Figures

2.1 Supplementary Figure S1

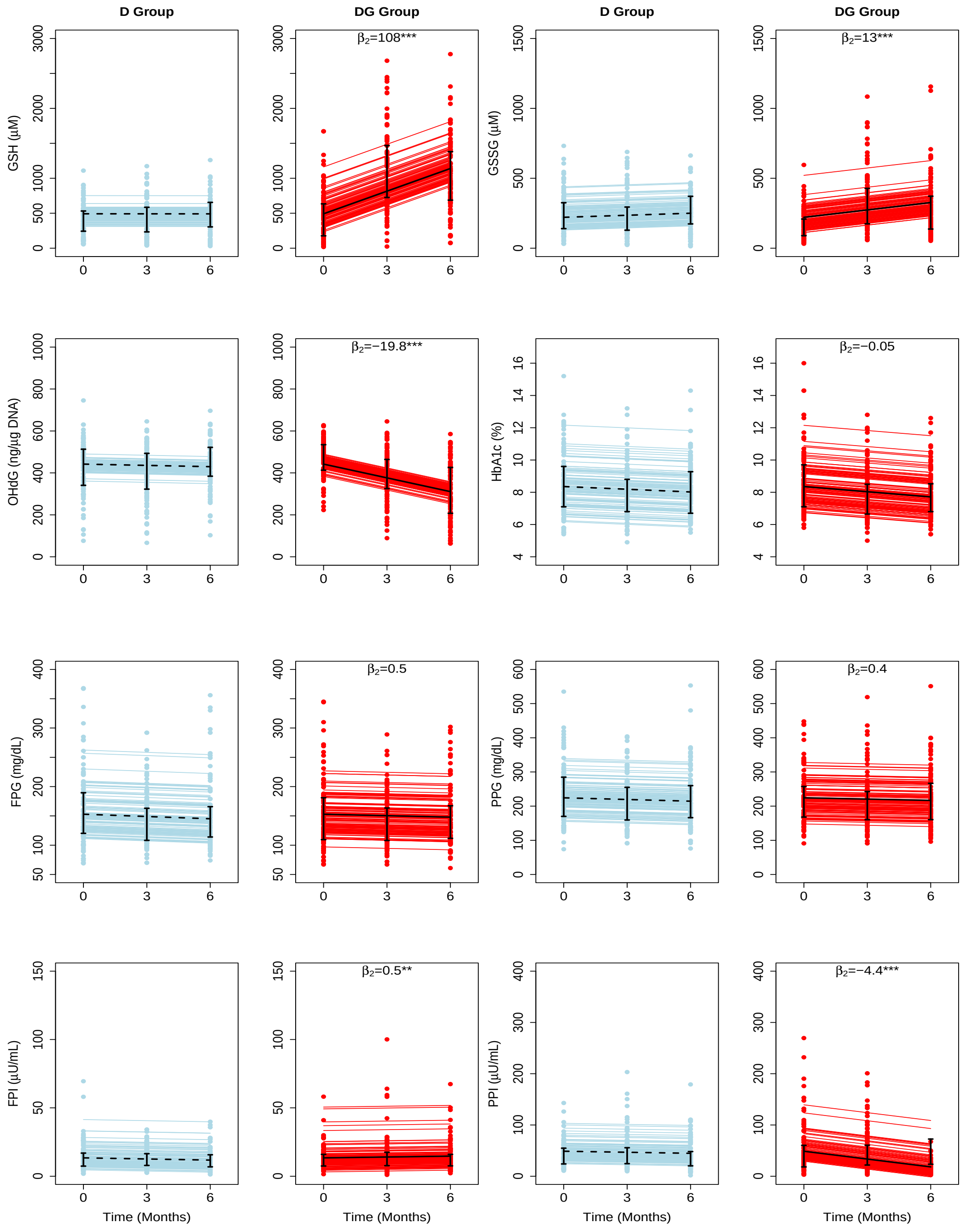

Supplementary Figure S1. Average treatment effects of GSH supplementation on biochemical changes estimated using LME Models. The fitted results of RIFS models for GSH, GSSG, 8-OHdG, HbA1c, FPG, FPI, PPG, and PPI variables in D group and DG groups (figure panels marked with titles D and DG) are overlaid here with the longitudinal data from 201 individuals (100 D subjects in blue circles, 101 DG subjects in red circles) at different visits. Solid blue and red lines depict the fitted subject-specific mean trajectories in the D group and the DG group, respectively. The black dotted and solid lines represent the group-wise means for D and DG, respectively. Interquartile ranges of the data for D and DG groups are shown with vertical interval plots (25th-75th quartiles) at each visit. The average treatment effects of GSH supplementation (β_2) are denoted on each panel corresponding to the DG group. The estimated β_2 was significant on the rate of changes in GSH ($\beta_2 = 108\mu M$ per month), GSSG ($\beta_2 = 13\mu M$ per month), 8-OHdG ($\beta_2 = -12.1 ng/\mu g$ DNA per month), FPI ($\beta_2 = 0.5\mu U/mL$ per month) and PPI ($\beta_2 = -4.4\mu U/mL$ per month) levels. The significance levels of parameter estimates are given by $^{*}p < 0.05$, $^{**}p < 0.01$, and $^{***}p < 0.001$. Abbreviations of the variables used here are the same as in Figure 1.

2.2 Supplementary Figure S2

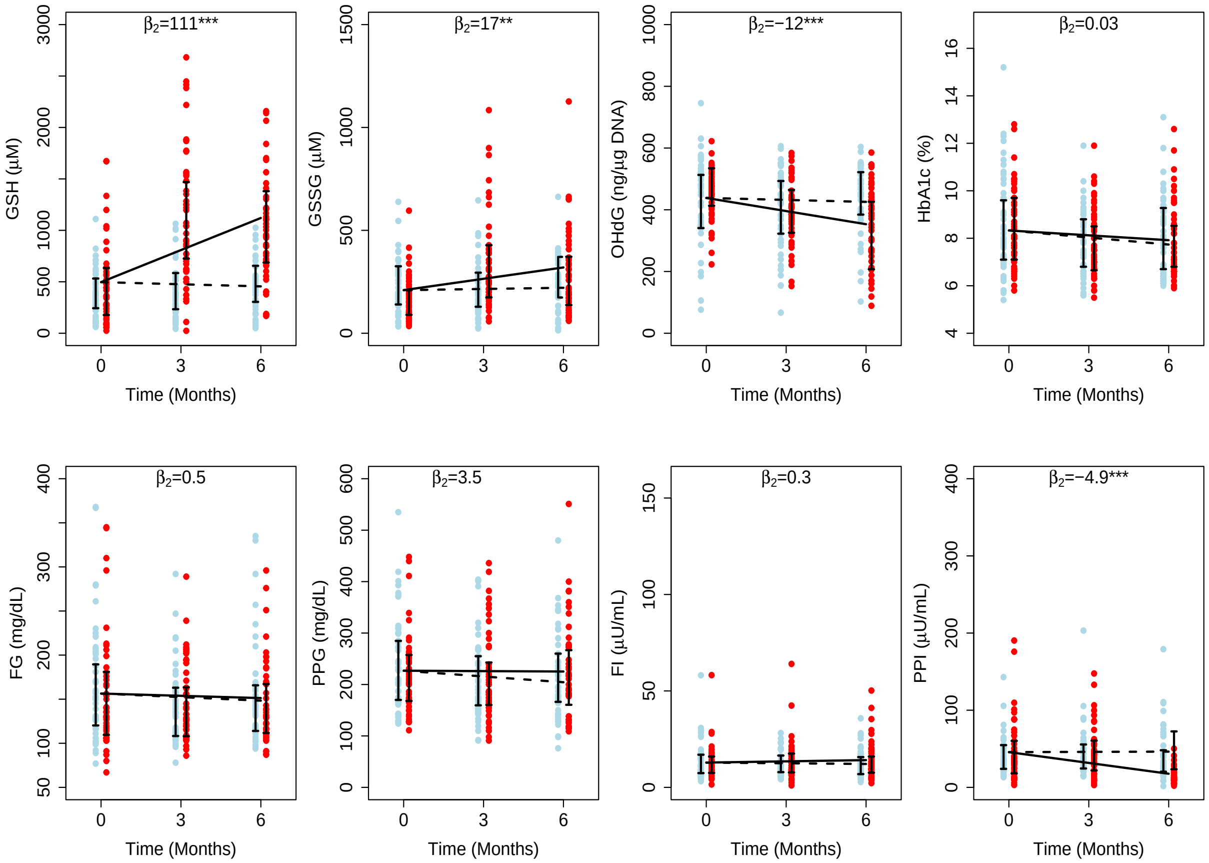

Supplementary Figure S2. Average treatment effects of GSH supplementation in younger diabetics using RIRS models. The fitted results of RIRS models for GSH, GSSG, 8-OHdG, HbA1c, FPG, FPI, PPG, and PPI variables of younger adults (YA) are shown on different panels here with the longitudinal data (blue circles for D individuals and red circles for DG individuals) at different visits. The data from 94 younger adults (48 from D and 46 from DG) are overlaid with group-wise mean trajectories for D and DG groups represented by black dotted lines and solid lines, respectively. Interquartile data ranges for individuals (from D and DG) are shown with vertical interval plots (25th-75th quartiles) at each visit. The average treatment effects of GSH supplementation (β_2) on the rate of changes (slope) denoted on top of corresponding panels which are significant on GSH($\beta_2 = 111\mu M$ per month), GSSG ($\beta_2 = 17\mu M$ per month), 8-OHdG ($\beta_2 = -12ng/\mu g$ DNA per month), and PPI ($\beta_2 = -4.9\mu U/mL$ per month) levels of younger adults. Significance of these parameter estimates are given by $^{*}p < 0.05$, $^{**}p < 0.01$, and $^{***}p < 0.001$. Abbreviations of the variables are the same as in Figure 1.

2.3 Supplementary Figure S3

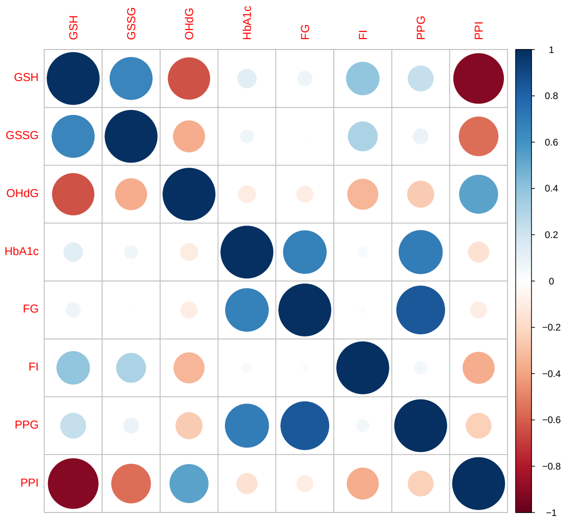

Supplementary Figure S3. Correlation diagram between subject-specific random slopes fitted for different outcome measures in younger subjects The strength and direction of correlation between subject-specific slopes fitted using RIRS models for different biochemical measures (GSH, GSSG, 8-OHdG, HbA1c, FPG, FPI, PPG, and PPI) are plotted here. The scales of Pearson's correlation coefficient have been classified as low ($r < 0.4$), moderate ($r < 0.6$), strong ($r > 0.6$), and very strong($r > 0.8$) correlations. Blue indicates a positive negative correlation, and red indicates a negative correlation.

2.4 Supplementary Figure S4

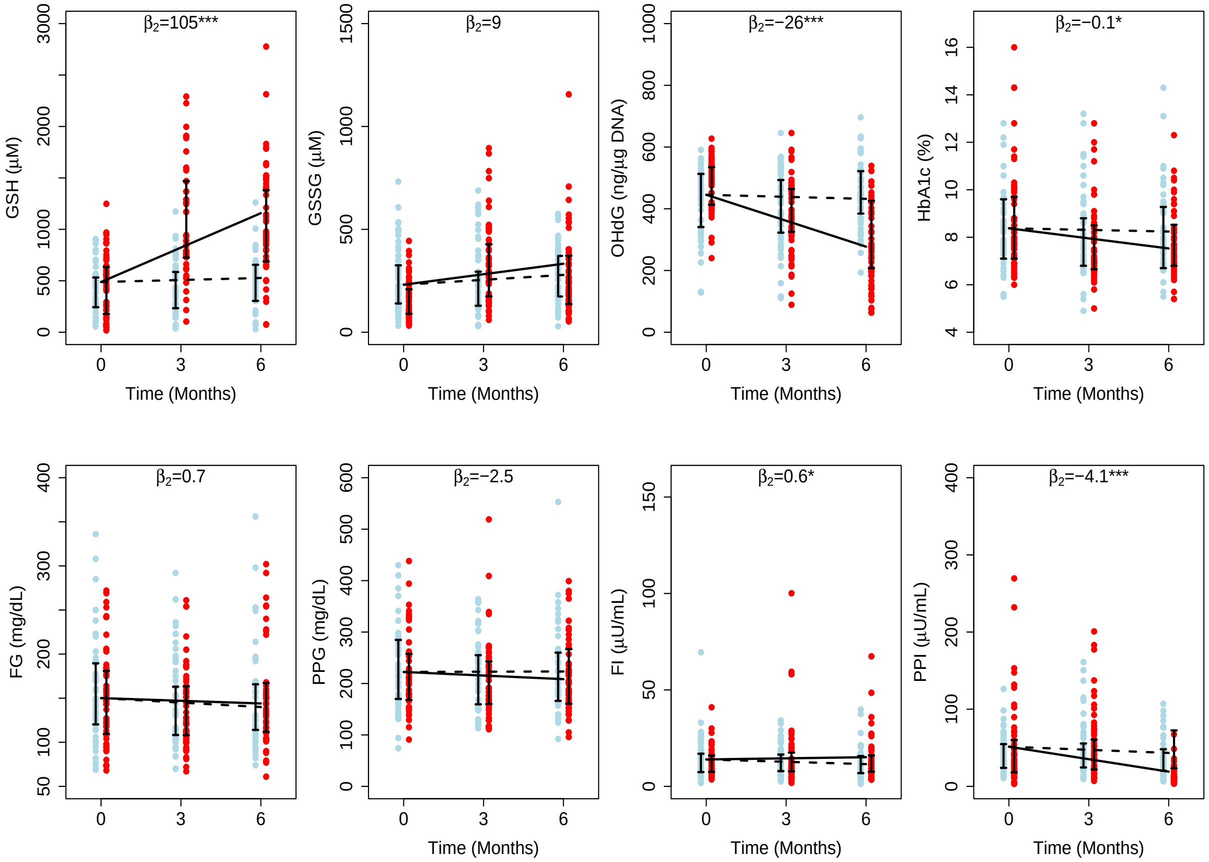

Supplementary Figure S4. Average treatment effects of GSH supplementation in elder diabetic adults using RIFS models. The fitted results of RIFS models for GSH, GSSG, 8-OHdG, HbA1c, FPG, FPI, PPG, and PPI variables of elder adults are shown on different panels here with the longitudinal data. The notations and abbreviations used here are the same as in Figure 1. Average treatment effects of GSH supplementation (β_2) estimated on the rate of changes (slope) are given on top of corresponding panels which are significant on GSH ($\beta_2 = 105\mu M$ per month), 8-OHdG ($\beta_2 = -26ng/\mu g$ DNA per month), HbA1c ($\beta_2 = -0.1\%$ per month), FPI ($\beta_2 = 0.6\mu U/mL$ per month), and PPI ($\beta_2 = -4.1\mu U/mL$ per month).

2.5 Supplementary Figure S5

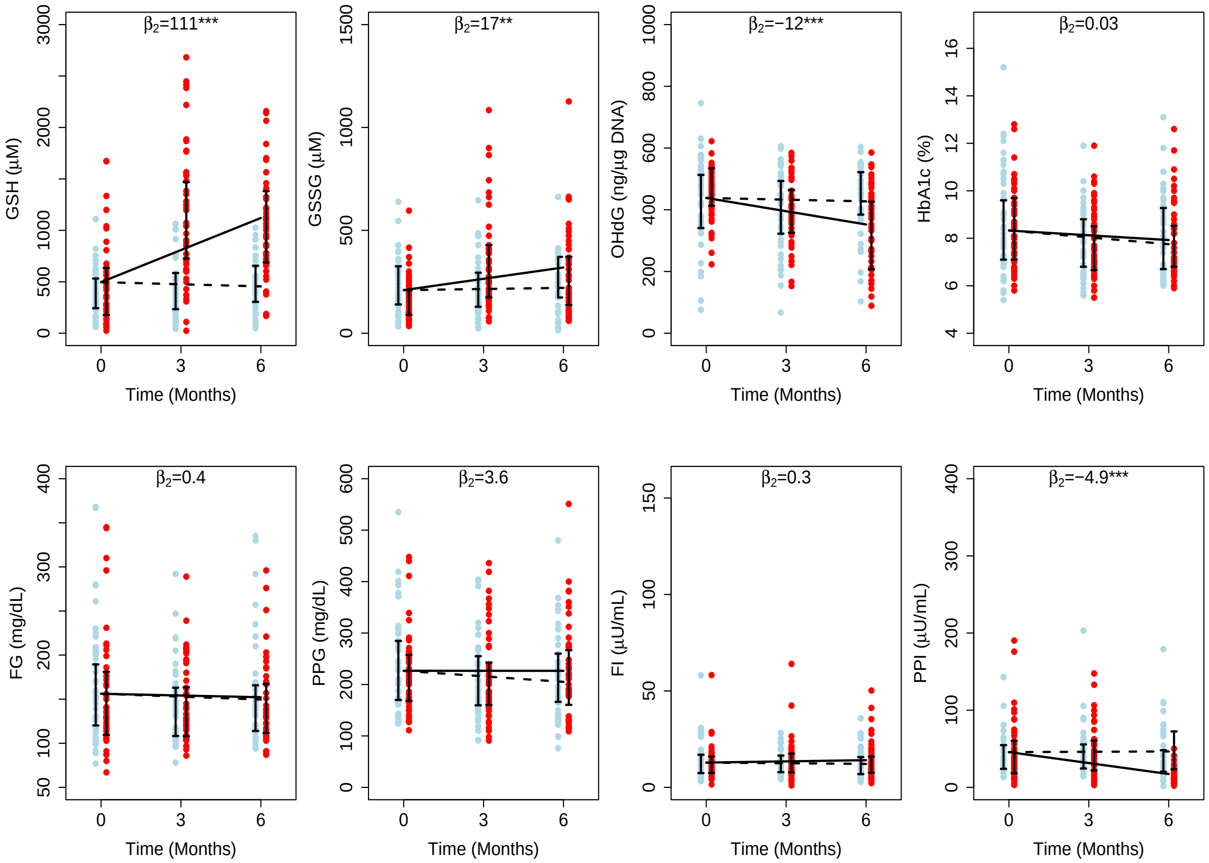

Supplementary Figure S5. Average treatment effects of GSH supplementation in younger diabetic adults using RIFS models. The fitted results of RIFS models for GSH, GSSG, 8-OHdG, HbA1c, FPG, FPI, PPG, and PPI variables of younger adults (YA) are shown on different panels here with the longitudinal data. The notations and abbreviations used here are the same as in Figure 1. Average treatment effects of GSH supplementation (β_2) estimated on the rate of changes (slope) are given on top of corresponding panels which are significant on GSH ($\beta_2 = 111\mu M$ per month), GSSG ($\beta_2 = 17\mu M$ per month), 8-OHdG ($\beta_2 = -12ng/\mu g$ DNA per month), and PPI ($\beta_2 = -4.9\mu U/mL$ per month).

2.6 Supplementary Figure S6

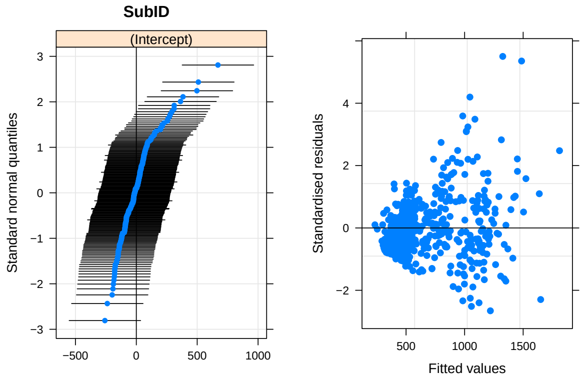

(A) GSH

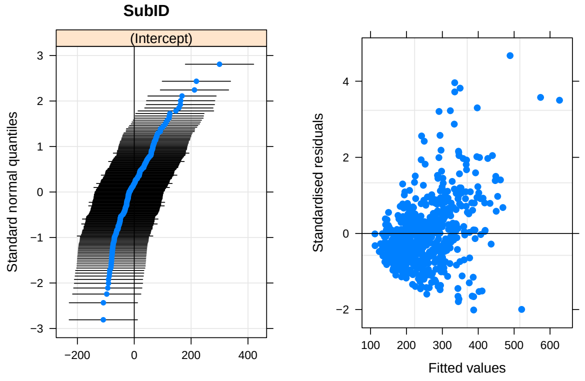

(B) GSSG

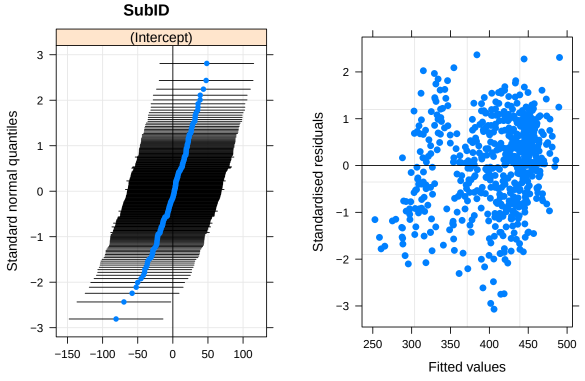

(C) 8-OHdG

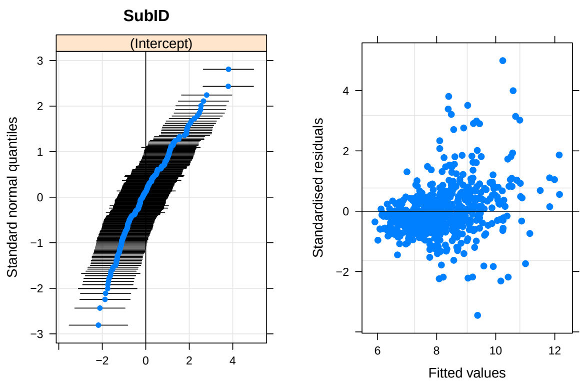

(C) HbA1c

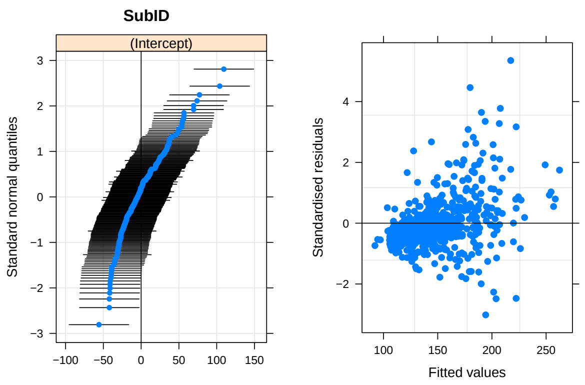

(D) FPG

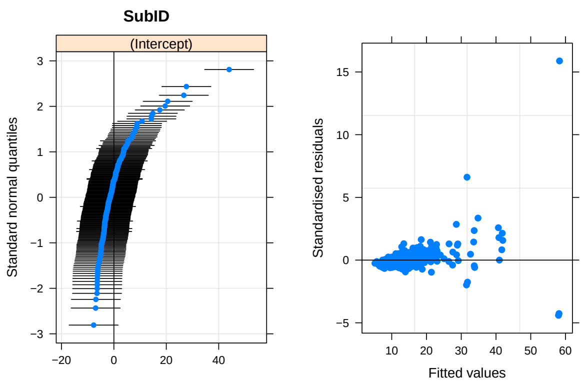

(E) FPI

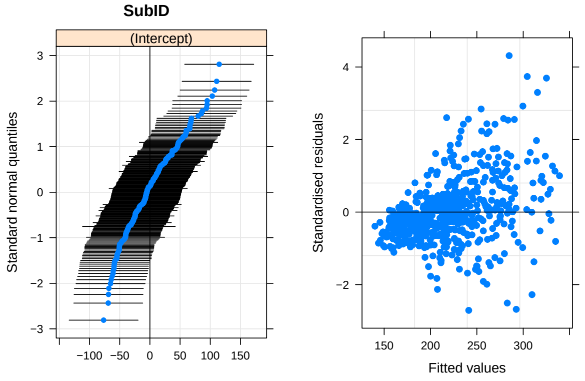

(F) PPG

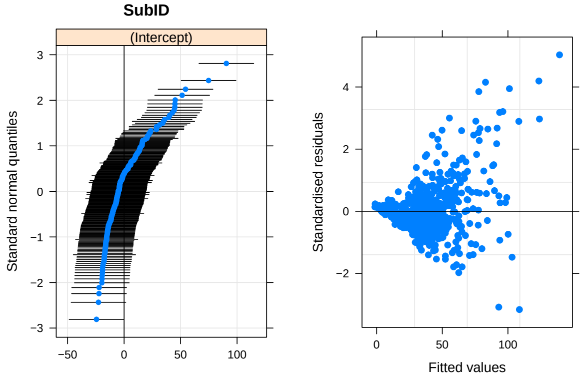

(G) PPI

Supplementary Figure S6. Model diagnostics plots of (A) GSH, (B) GSSG, (C) 8-OHdG, (D) HbA1c, (E) FPG, (F) FPI, (G) PPG, and (H) PPI obtained by fitting RIFS models are described here. The distribution of subject-specific random effects (intercepts) is plotted on the left panel. After fitting the models for each variable, the residuals are plotted on the right panels. Abbreviations of the variables used here are HbA1c—glycated hemoglobin, GSH—reduced glutathione, GSSG-oxidized glutathione, PP glucose—postprandial glucose, PP insulin—postprandial insulin, and 8-OHdG - 8-hydroxy-2-deoxy guanosine.

2.7 Supplementary Figure S7

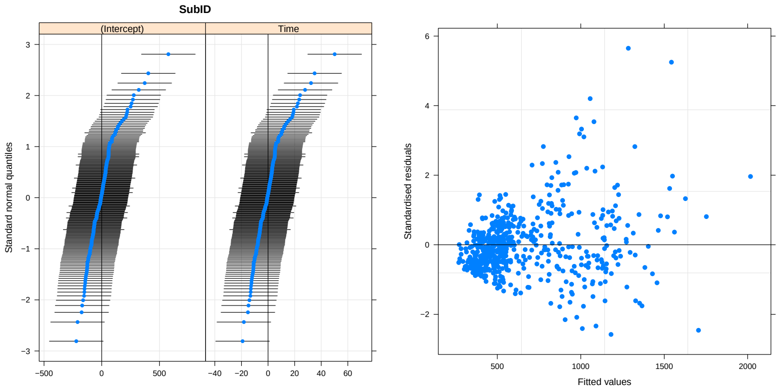

(A) GSH

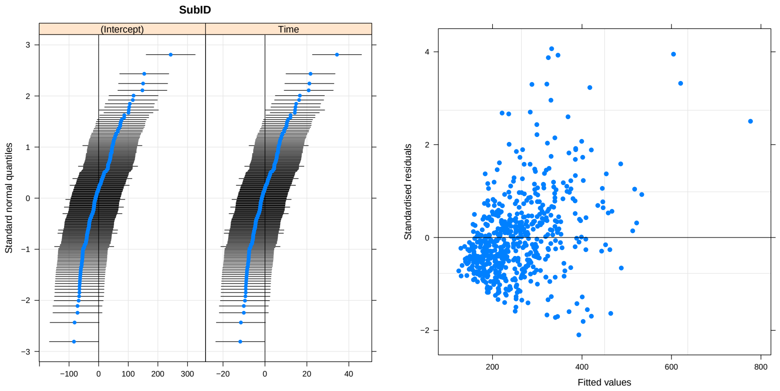

(B) GSSG

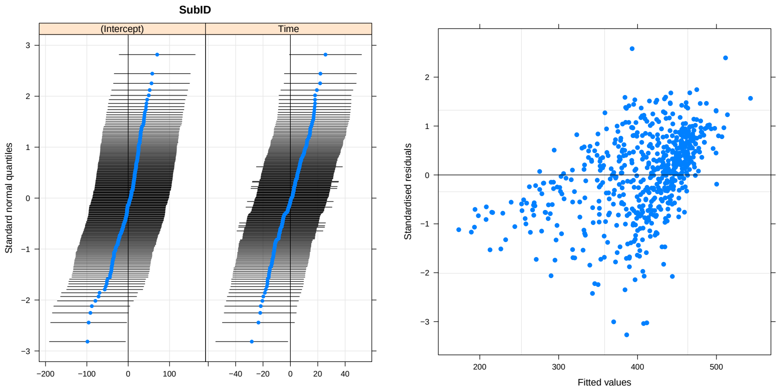

(C) 8-OHdG

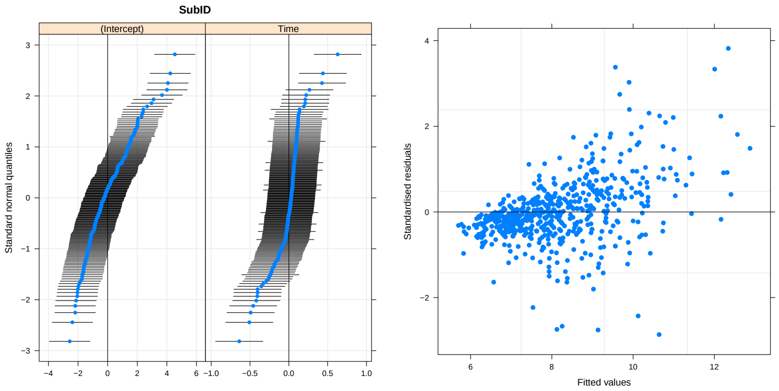

(D) HbA1c

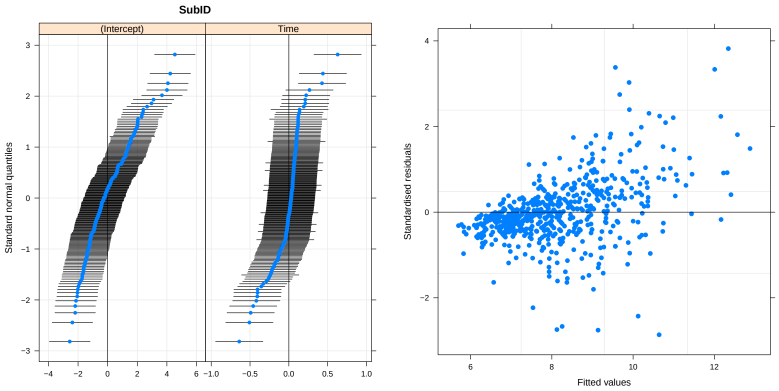

(E) FPG

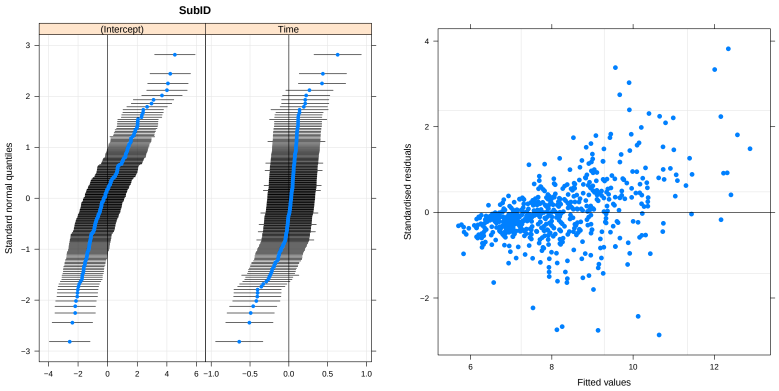

(F) FPI

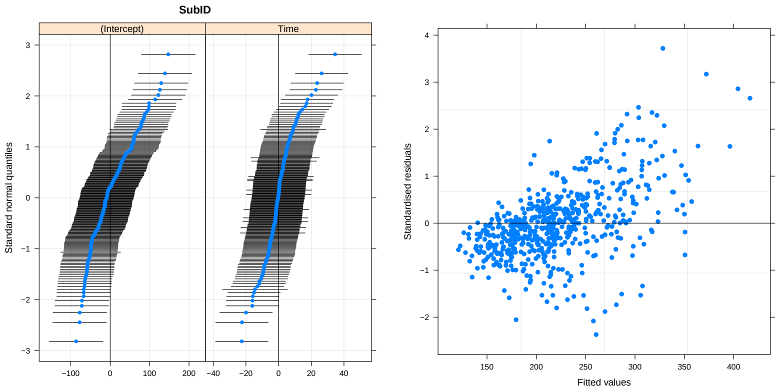

(G) PPG

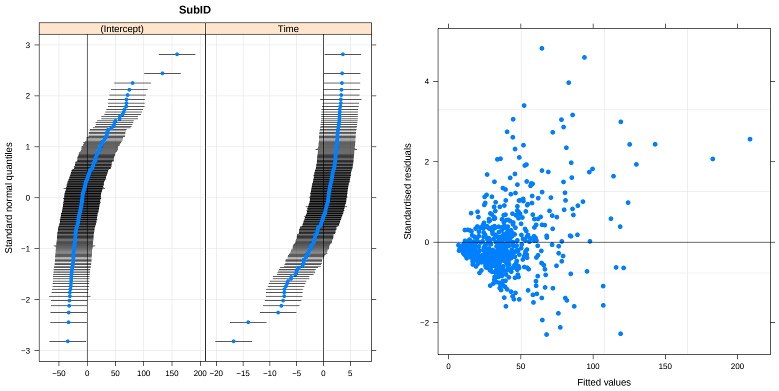

(H) PPI

Supplementary Figure S7. Model diagnostics plots of (A) GSH, (B) GSSG, (C) 8-OHdG, (D) HbA1c, (E) FPG, (F) FPI, (G) PPG, and (H) PPI obtained by fitting RIRS models are described here. The distribution of subject-specific random effects (intercepts and slopes) is plotted on the left panels. After fitting the models for each variable, the residuals are plotted on the right panels. Abbreviations of the variables used here are the same as in Figure 1.

3 Supplementary Tables

3.1 Supplementary Table S1

Subject ID	Time	Groups	Age	Y (eg: GSH in μM)
1	0	0	56	408
1	3	0	56	860
1	6	0	56	608
..	..	..	..	..
..	..	..	..	..
102	0	0	48	434
102	3	0	48	655
102	6	0	48	533
103	0	1	55	..
103	3	1	55	..
103	6	1	55	..
..	..	..	..	..
..	..	..	..	..
206	0	1	47	..
206	3	1	47	..
206	6	1	47	..

Supplementary Table S1. The sample structure of the data from the D and DG groups in the clinical trial is described here. The data consists of measurements from 102 subjects in D and 104 subjects in DG on three visits during the study period (0, 3, and 6 months of the study). The Group IDs for subjects in D and DG are encoded as 0 and 1. The models are fitted using these data sets.

3.2 Supplementary Table S2

Results from RIRS models are shown below.

Variable	Fixed effect parameters		
	β_0 (SE)	β_1 (SE)	β_2 (SE)
GSH	492.2(27.4)***#	0.04 (8.6)	107.7(10.3)***#
GSSG	221(10.1)***#	4.7 (3.3)	13.02(4.2)***#
8-OHdG	442.02(7.5)***#	-2.8 (2.6)	-18.5(2.9)***#
HbA1c	8.4(0.1)***#	-0.06 (0.03)	-0.05 (0.04)
FPG	152.9(3.9)***#	-1.33 (1.09)	0.4 (1.3)
FPI	13.4(0.66)***#	-0.3(0.14)*	0.5(0.2)**
PPG	224.4(5.4)***#	-1.6 (1.6)	0.3 (2)
PPI	48.8(2.8)***#	-0.8 (0.6)	-4.1(0.6)***#

(A) Fixed-effects parameters

Variable	Random effect parameters			
	σ_0	σ_1	σ_{01}	σ_e
GSH	162.3	14.1	2286.3	386.8
$GSSG$	66.3	9.4	621.3	139.6
$8-OHdG$	55.95	17.04	-551.2	98.5
$HbA1c$	1.53	0.21	-0.17	0.96
FPG	46.07	8.71	-215.2	33.2
FPI	7.8	0.07	0.53	5.7
PPG	59.68	11.38	-296	51.6
PPI	32.54	3.31	-107.9	23.8

(B) Random-effects parameters

Supplementary Table S2. The estimates of fixed effects (A) and random effects (B) parameters obtained by fitting RIRS models are given in the table. Abbreviations of the variables used here are HbA1c-glycated hemoglobin, GSH—reduced glutathione, GSSG-oxidized glutathione, PP glucose—postprandial glucose, PP insulin—postprandial insulin, and 8-OHdG-8-hydroxy-2-deoxy guanosine. Significance of parameter estimates are given by $^{*}p < 0.05$, $^{**}p < 0.01$, and $^{***}p < 0.001$. # symbols are marked for significance levels retained after the corrections for multiple comparisons.

3.3 Supplementary Table S3

Results from RIFS models are shown below.

Variable	Fixed effect parameters		
	β_0 (SE)	β_1 (SE)	β_2 (SE)
GSH	492.2(28.9)***#	-0.2 (8.4)	108.2(9.8)***#
GSSG	221(11.3)***#	4.9 (3.1)	12.7(3.8)***#
8-OHdG	441.9(7.7)***#	-2.04 (2.3)	$-19.8(2.6)^{***}$#
HbA1c	8.4(0.1)***#	-0.06 (0.03)	-0.05 (0.04)
FPG	152.9(3.7)***#	-1.3 (0.95)	0.46 (1.2)
FPI	13.4(0.7)***#	-0.3 (0.13)	0.48(0.2)**#
PPG	224.4(5.4)***#	-1.6 (1.4)	0.37 (1.7)
PPI	48.8(2.3)***#	-0.7 (0.6)	$-4.4(0.7)^{***}$#

(A) Fixed-effects parameters

Variable	Random-effects parameters	
	σ_0	σ_e
GSH	200.9	389.9
GSSG	91.82	142.8
8-OHdG	40.35	110.6
HbA1c	1.27	1.15
FPG	35.95	42.17
FPI	8.01	5.7
PPG	50.68	61.72
PPI	22	25.86

(B) Random-effects parameters

Supplementary Table S3. The estimated fixed effects (A) and random effects (B) parameters obtained by fitting RIFS models are given in the table. Abbreviations of the variables used here are the same as in Supplementary Table S2. Significance of parameter estimates are given by $^{*}p < 0.05$, $^{**}p < 0.01$, and $^{***}p < 0.001$. # symbols are marked for significance levels retained after the corrections for multiple comparisons.

3.4 Supplementary Table S4

Results from independent LME models for analyzing the effects on elder and younger diabetic subgroups are shown below.

Variable	Fixed effect parameters (EA)			Fixed effect parameters (YA)		
	β_0 (SE)	β_1 (SE)	β_2 (SE)	β_0 (SE)	β_1 (SE)	β_2 (SE)
GSH	488.1(33.4)***#	6.5 (11.5)	104(14.05)***#	496.6(44.9)***#	-6.9 (13.07)	111(15.3)***#
GSSG	231(14.5)***#	7.6 (4.5)	9.5 (5.6)	209.4(14.03)***#	1.9 (4.9)	16.5(6.2)**
8-OHdG	445.3(10.2)***#	-3.3 (3.5)	-23.7(3.9)***#	438.3(11)***#	-2.1 (3.6)	-12.06(4.2)**#
HbA1c	8.4(0.2)***#	-0.02 (0.04)	-0.1(0.05)*	8.3(0.2)***#	-0.1(0.04)*	0.03(0.06)
FPG	150.1(5.2)***#	-1.7 (1.2)	0.6 (1.6)	156.4(5.9)***#	-1.3 (1.8)	0.5 (2.1)
FPI	14(1.002)***#	-0.4 (0.2)	0.6(0.3)*	12.9(0.9)***#	-0.12 (0.15)	0.3(0.2)
PPG	222.5(6.9)***#	0.14 (1.97)	-2.5 (2.4)	226.8(8.3)***#	-3.7 (2.5)	3.5 (3.1)
PPI	51.4(4.3)***#	-1.6(0.82)*	-3.6(0.77)***#	45.7(3.3)***#	0.1 (0.7)	-4.8(0.9)***#

Supplementary Table S4A. Comparison between the estimates of fixed-effect parameters in the RIRS model for individuals in different age classes The fixed-effects parameter values obtained by fitting the data from the subgroup of elder adults (EA) and younger adults (YA) separately using the RIRS models for GSH, GSSG, 8-OHdG, HbA1c, FPG, FPI, PPG, and PPI variables are shown here with the standard errors (SE). Average treatment effects (β_2) of GSH supplementation are observed to be significant on GSH, 8-OHdG, HbA1c, FPI and PPI levels for EA and on GSH, GSSG, 8-OHdG and PPI levels for YA. Significance of parameter estimates are given by $^{*}p < 0.05$, $^{**}p < 0.01$, and $^{***}p < 0.001$. # symbols are marked for significance levels retained after the corrections for multiple comparisons.

Variable	Fixed effect parameters (EA)			Fixed effect parameters (YA)		
	β_0 (SE))	β_1 (SE)	β_2 (SE)	β_0 (SE)	β_1 (SE)	β_2 (SE)
GSH	488.1(37.1)***#	6.5 (10.8)	105(12.6)***#	496.6(45)***#	-6.9 (13.06)	110.8(15.3)***#
GSSG	230.9(15.6)***#	7.9 (4.3)	9.04 (5.2)	209.5(16.06)***#	1.8 (4.5)	16.5(5.4)**
8-OHdG	445.2(10.3)***#	-2.2 (3.5)	-25.8(3.5)***#	438.3(11.2)***#	-1.8 (3.3)	-12.5(3.8)**#
HbA1c	8.4(0.2)***#	-0.02 (0.04)	-0.1(0.05)**	8.3(0.2)***#	-0.1(0.04)*	0.03(0.05)
FPG	150.1(4.9)***#	-1.7 (1.1)	0.7 (1.5)	156.2(5.6)***#	-1.1 (1.5)	0.4 (1.9)
FPI	14(1.002)***#	-0.4 (0.2)	0.6(0.3)*	12.9(0.9)***#	-0.12 (0.14)	0.3(0.2)
PPG	222.5(6.8)***#	0.1 (1.8)	-2.5 (2.2)	226.6(8.3)***#	-3.6 (2.1)	3.5 (2.7)
PPI	51.4(3.4)***#	-1.3 (0.9)	-4.1(1.07)***#	45.7(3)***#	0.1 (0.8)	-4.9(1)***#

Supplementary Table S4B. Comparison between the estimates of fixed-effect parameters in the RIFS model for individuals in different age classes The fixed-effects parameter values were obtained by fitting the data from the subgroup of elder adults (EA) and younger adults (YA) separately using the RIFS models for GSH, GSSG, 8-OHdG, HbA1c, FPG, FPI, PPG, PPI variables are shown here with the standard errors (SE). Average treatment effects (β_2) of GSH supplementation are observed to be significant on GSH, 8-OHdG, HbA1c, FPI, and PPI levels for EA and on GSH, GSSG, 8-OHdG, and PPI levels for YA. Significance of parameter estimates are given by $^{*}p < 0.05$, $^{**}p < 0.01$, and $^{***}p < 0.001$. # symbols are marked for significance levels retained after the corrections for multiple comparisons.

3.5 Supplementary Table S5

Results from different LME models for analyzing the age effects are shown below.

Variable	Fixed effect parameters		
	β_0 (SE)	β_1 (SE)	β_2 (SE)
GSH	492.1(27.5)***#	3.4 (8.5)	1.9(0.2)***#
GSSG	221(10.1)***#	5.1 (3.2)	0.2(0.07)**
8-OHdG	442(7.5)***#	-2.5 (2.5)	$-0.3(0.05)^{***}$#
HbA1c	8.4(0.1)***#	-0.05 (0.03)	-1.05 (0.0006)
FPG	152.9(3.9)***#	-1.1 (1.07)	2(0.02)
FPI	13.4(0.7)***#	$-0.3(0.13)^{*}$	0.008(0.003)**
PPG	224.4(5.4)***#	-1.2 (1.5)	-0.009 (0.03)
PPI	48.8(2.8)***#	-1 (0.6)	$-0.07(0.01)^{***}$#

(A) Results obtained by fitting with Model 2

Variable	Fixed effect parameters			
	β_0 (SE)	β_1 (SE)	β_2 (SE)	β_3 (SE)
GSH	492.2(27.4)***#	-0.03 (8.6)	82.1(38.5)*	0.5 (0.7)
GSSG	221(10.1)***#	4.7 (3.3)	9.7(15.6)	0.06 (0.3)
8-OHdG	442(7.5)***#	-2.7 (2.5)	3.9(10.8)***#	$-0.4(0.2)^{*}$
HbA1c	8.4(0.1)***#	-0.06 (0.03)	0.1 (0.14)	-0.003 (0.002)
FPG	152.9(3.9)***#	-1.3 (1.1)	5.5 (4.9)	-0.09 (0.09)
FPI	13.4(0.7)***#	$-0.3(0.13)^{*}$	0.2 (0.7)	0.005 (0.01)
PPG	224.4(5.4)***#	-1.6 (1.6)	10.7 (7.3)	-0.2 (0.1)
PPI	48.8(2.8)***#	-0.8 (0.6)	-3.8 (2.1)	-0.007 (0.04)

(B) Results obtained by fitting with Model 3

Variable	Fixed effect parameters			
	β_0 (SE)	β_1 (SE)	β_2 (SE)	β_3 (SE)
GSH	492.2(27.4)***#	0.08 (8.6)	110.8(12.2)***#	104(12.9)***#
GSSG	221(10.1)***#	4.7 (3.3)	13.7(4.9)**	12.2(5.2)*
8-OHdG	442(7.5)***#	-2.6 (2.5)	$-24.5(3.4)^{***}$#	$-12.2(3.6)^{***}$#
HbA1c	8.4(0.1)***#	-0.06 (0.03)	-0.08 (0.04)	-0.01 (0.05)
FPG	152.9(3.9)***#	-1.3 (1.09)	-0.19 (1.6)	1.1 (1.7)
FPI	13.4(0.7)***#	$-0.3(0.1)^{*}$	0.4(0.2)*	0.5(0.2)*
PPG	224.4(5.4)***#	-1.6 (1.6)	-1 (2.3)	1.7 (2.4)
PPI	48.8(2.8)***#	-0.8 (0.6)	$-4.1(0.7)^{***}$#	$-4.2(0.7)^{***}$#

(C) Results obtained by fitting with Model 4

Variable	AIC				BIC			
	Model 1	Model 2	Model 3	Model 4	Model 1	Model 2	Model 3	Model 4
GSH	8870.5	8874.5	8872	8872.3	8901.2	8905.2	8907.1	8907.3
GSSG	7718.1	7718.4	7720	7720	7748.8	7749.1	7755.1	7755.1
8-OHdG	7331.1	7326.6	7328.5	7323.8	7361.8	7357.3	7363.6	7358.9
HbA1c	2149	2148.2	2149.7	2149.3	2179.7	2178.9	2184.8	2184.4
FPG	6323	6323.1	6323.9	6324.5	6353.7	6353.8	6359	6359.6
FPI	4139.6	4139.5	4141.4	4141.5	4170.3	4170.1	4176.4	4176.6
PPG	6740.8	6740.8	6740.6	6741.8	6771.5	6771.4	6775.6	6776.9
PPI	5708.9	5712	5710.9	5710.9	5739.6	5742.6	5745.9	5745.9

(D) Comparison between four models to study age-effects (Model 1, Model 2, Model 3, model4)

Supplementary Table S5. Fitted results of new candidate LME models (A) Model 2, (B) Model 3, (C) Model 4, to study the age effects on GSH supplementation are shown here with their (D) model comparisons with our original RIRS model (Model 1, without age included as a model variable). Abbreviations of the variables and the significance levels used here are the same as in Table S2. Fits from four models are compared using their AIC and BIC estimates, and Model 1 was found to be the better-fit model across all eight endpoints.
